# Anatomy and Physiology of the Digestive Tract of *Drosophila melanogaster*

**DOI:** 10.1534/genetics.118.300224

**Published:** 2018-10-01

**Authors:** Irene Miguel-Aliaga, Heinrich Jasper, Bruno Lemaitre

**Affiliations:** * Medical Research Council London Institute of Medical Sciences, Imperial College London, W12 0NN, United Kingdom; † Buck Institute for Research on Aging, Novato, California 94945-1400; ‡ Immunology Discovery, Genentech, Inc., San Francisco, California 94080; § Global Health Institute, School of Life Sciences, École polytechnique fédérale de Lausanne, CH-1015 Lausanne, Switzerland

**Keywords:** FlyBook, *Drosophila*, intestine, midgut, enteric nervous system, microbiota, immunity, metals, aging, digestion, absorption, enteroendocrine, stem cells

## Abstract

The gastrointestinal tract has recently come to the forefront of multiple research fields. It is now recognized as a major source of signals modulating food intake, insulin secretion and energy balance. It is also a key player in immunity and, through its interaction with microbiota, can shape our physiology and behavior in complex and sometimes unexpected ways. The insect intestine had remained, by comparison, relatively unexplored until the identification of adult somatic stem cells in the *Drosophila* intestine over a decade ago. Since then, a growing scientific community has exploited the genetic amenability of this insect organ in powerful and creative ways. By doing so, we have shed light on a broad range of biological questions revolving around stem cells and their niches, interorgan signaling and immunity. Despite their relatively recent discovery, some of the mechanisms active in the intestine of flies have already been shown to be more widely applicable to other gastrointestinal systems, and may therefore become relevant in the context of human pathologies such as gastrointestinal cancers, aging, or obesity. This review summarizes our current knowledge of both the formation and function of the *Drosophila melanogaster* digestive tract, with a major focus on its main digestive/absorptive portion: the strikingly adaptable adult midgut.

CONTROL mechanisms are key to animal survival; they ensure stability and can also drive adaptive change. It is relatively straightforward for animals to keep their internal environment under check, but their homeostasis is also critically dependent on a fluctuating external environment over which they have much less control. By capturing part of their immediate external environment inside the lumen of their digestive tract, however, animals were provided with an excellent opportunity to sense and react to their (now ingested) outside world; to transform and extract what they need from it, and to mount defense responses against it if necessary. It is therefore not surprising that digestive tracts, including those of insects, are complex and remarkably plastic organs. It is somewhat more surprising that it took so long for the community of *Drosophila* researchers to “discover” the digestive tract of their fruit flies. Once they did, however, they exploited its genetic amenability in powerful and creative ways that have shed light on broader biological questions around stem cells and their niches, interorgan signaling and immunity. In the following sections, we summarize our current knowledge of the development and physiology of the *Drosophila melanogaster* digestive tract, with a major focus on its main digestive/absorptive portion: the strikingly adaptable adult midgut.

## Structure of the Digestive Tract

The *Drosophila* intestine is a complex organ consisting of multiple cell types of heterogeneous developmental origin. While it may be unsurprising that its muscles, neurons, and tracheal supply arise from cell clusters located in different embryonic territories, even its epithelial lining originates from two different germ layers and three distinct sites in the embryo. The behavior of its different cell types can also differ quite dramatically during the transition from larval to adult life (ranging from apoptosis to persistence without remodeling). Partly as a result of these heterogeneous origins and complex developmental trajectory, the adult intestine is a regionalized and plastic organ, and some of its portions can undergo striking remodeling throughout adult life. This section describes both the development and adult structure of the *Drosophila* intestine, with a focus on the midgut: the major site of digestion and absorption, as well as the main focus of scientific interest in the past decade.

### Embryonic and larval development

**Figure 1:**
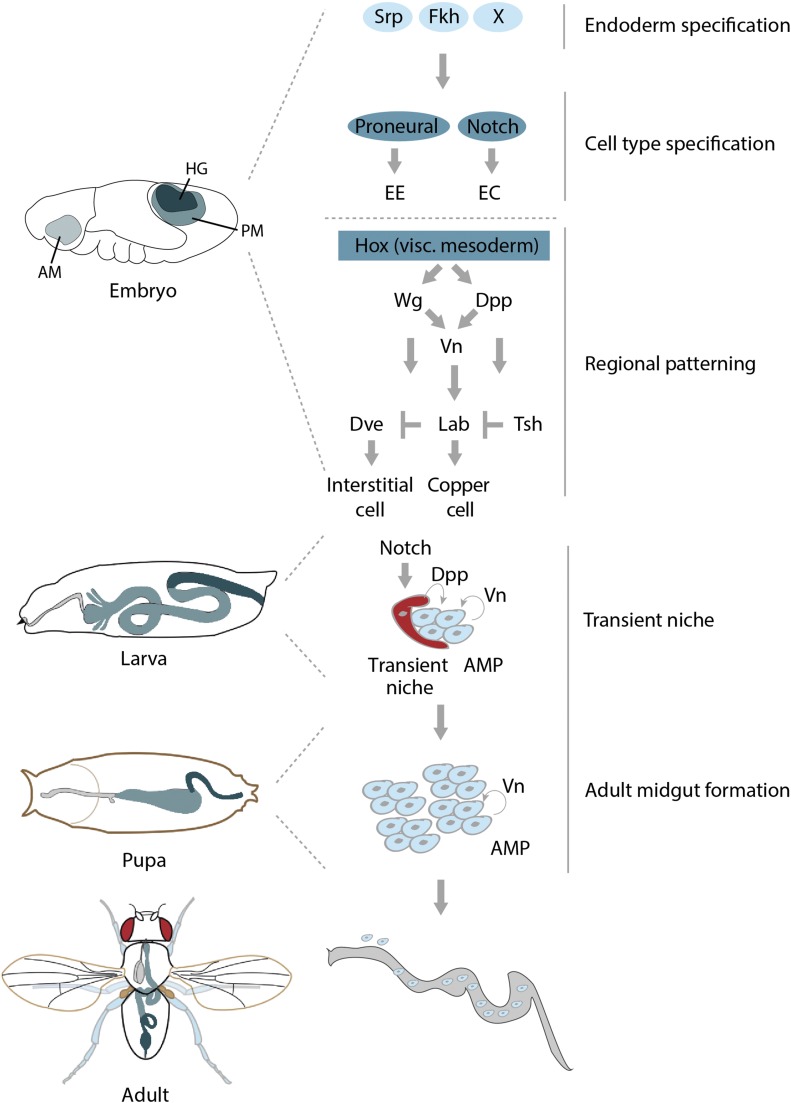
Developmental transitions and key factors in intestinal cell fate decisions. See section *Embryonic and larval development* for details.

Figure 1 illustrates key developmental transitions and mediators. As opposed to the foregut and hindgut, which are of ectodermal origin, the *Drosophila* midgut originates from the endoderm and is thus established during gastrulation. After induction of the endodermal fate by maternal factors, endoderm is further specified by several transcription factors that are widely conserved in evolution, including the GATA transcription factor Serpent (Srp) and the HNF/Fork Head (Fkh) transcription factors (Takashima *et al.* 2013). Endodermal cells will then undergo specification into either enterocyte (EC)-like or enteroendocrine (EE)-like cells through the action of proneural proteins (such as Lethal of scute, which promotes endocrine fates) and Notch signaling (activation of Notch promotes EC fates) (Takashima *et al.* 2011a, 2013). The balance between proneural protein activity and Notch signaling activity will thus ultimately determine the cellular composition of the midgut, yet the upstream regulators of proneural gene expression (in addition to GATA and Fkh transcription factors) remain largely unknown (Takashima *et al.* 2011a, 2013).

Extracellular signals derived from the adhering visceral mesoderm then promote differentiation of the midgut endoderm around stage 16 [for reviews see Bienz (1997), Nakagoshi (2005)]. The four posterior Homeobox (Hox) genes in the visceral mesoderm promote the expression of signaling molecules that specify the subdivision of the midgut endoderm along its anterior-posterior axis [for reviews see Bienz (1997), Miller *et al.* (2001a,b)]. These factors include Decapentaplegic (Dpp), a member of the Bone morphogenetic protein (BMP)/Transforming growth factor β (Tgfβ) superfamily, and Wingless/Wnt (Wg), which in turn induce the expression of Vein, a ligand for the EGF receptor, in the visceral mesoderm (Immerglück *et al.* 1990; Reuter and Scott 1990). All three signaling molecules are involved in the induction of morphogenetic events that subdivide the midgut (Immerglück *et al.* 1990; Reuter and Scott 1990; Casas-Tinto *et al.* 2008). In parasegment 7 of the endoderm, they induce, for example, *labial* (*lab*): a gene coding for a Hox protein required for endoderm differentiation (Immerglück *et al.* 1990; Reuter and Scott 1990; Casas-Tinto *et al.* 2008).

Complex interactions between Lab and other transcription factors induced by Dpp and Wg further shape the midgut. *teashirt* (*tsh*) negatively regulates *lab* and is required for interstitial cell precursors (Mathies *et al.* 1994), whereas *defective proventriculus* (*dve*) is broadly expressed in midgut precursor cells and is later repressed by *lab* (Nakagoshi *et al.* 1998). Dpp is believed to form a morphogenetic gradient that induces the high-threshold target *lab* and the low-threshold target *dve* in different fields of the gradient, resulting in the specification of two different types of ECs: copper cells (Lab-positive) and interstitial cells (Dve-positive), respectively (Nakagoshi 2005).

In addition to the formation of the larval midgut, endodermal progenitors for the adult midgut are also formed in the early embryo. These cells, adult midgut progenitors (AMPs), form small clusters of proliferating, undifferentiated cells that are attached to the basal surface of the larval gut epithelium. During metamorphosis, AMPs form the adult midgut by dispersing and proliferating within distinct islands, in a process that is regulated by Epidermal growth factor receptor (Egfr) signaling (Jiang and Edgar 2009). AMPs are regulated by a transient niche in the larval midgut that is established through Notch signaling and maintains AMPs in an undifferentiated state through Dpp signaling (Mathur *et al.* 2010). Niche cells go on to differentiate during metamorphosis, spreading out between the newly forming adult gut and the degenerating larval midgut and forming a transient pupal epithelium. At that stage, AMPs form large cell clusters that eventually fuse to make the adult midgut epithelium (Takashima *et al.* 2011b). Degeneration of the larval midgut requires activation of autophagy rather apoptosis (Denton *et al.* 2009), and is modulated by Dpp, the class I phosphoinositide-3-kinase pathway and ecdysone (Denton *et al.* 2012, 2018). The physiological role of the transient pupal epithelium remains to be established and will be of interest for future work.

### The adult gut and its cell types: genetic and anatomical compartmentalization

**Figure 2:**
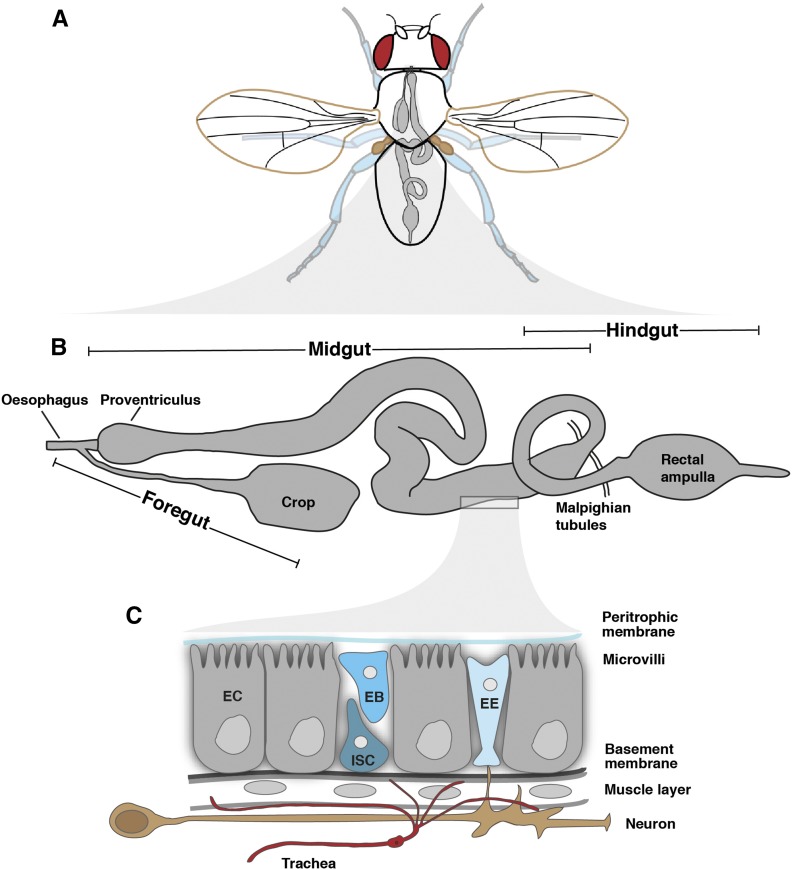
The adult intestine and its cell types. (A) The digestive tract is highlighted in gray inside an adult fly. (B) Main anatomical features of the adult digestive tract. (C) General cellular composition of the digestive tract. See section *The adult gut and its cell types: genetic and anatomical compartmentalization* for details.

As shown in Figure 2C, the “ground plan” of the adult *Drosophila* gut consists of a tube lined by an epithelial monolayer consisting of four cell types: intestinal stem cells (ISCs), absorptive ECs, secretory EE cells, and enteroblasts (EBs): a postmitotic, immature cell type which will differentiate as an EC (or, possibly, as an EE, see below for current view of lineage relationships). Of note, midgut epithelial cells have a reverse arrangement of junctions compared to other *Drosophila* epithelia, with occluding junctions above *adherens* junctions, as in vertebrates (Chen *et al.* 2018a). This epithelium is surrounded by visceral muscle and protected toward the lumen by secreted mucus and, posterior to the foregut, by a chitinous layer: the peritrophic matrix (Hegedus *et al.* 2009). There are, however, substantial variations of this common theme, both at the gross anatomy and cellular levels. These are primarily determined by the developmental origin of a given gut region, as well as its specific location along the antero-posterior axis. Anatomical specializations and regional compartmentalization both enable sequential ingestion, storage, digestion, absorption, and defecation (Karasov *et al.* 2011).

Anteriorly, the ectodermally derived foregut is subdivided into esophagus, crop, and cardia (Figure 2B). The crop is a diverticulated structure unique to Diptera, consisting of a complex array of valves and sphincters ensuring transit of intestinal contents in and out of the crop into the main alimentary canal. Although its functions in *Drosophila* remain to be investigated, work in other insects suggests that it may function in early digestion, detoxification, microbial control, and/or food storage (Stoffolano and Haselton 2013). The cardia (also known as proventriculus) is a complex bulb-shaped organ composed of three epithelial layers. It produces the peritrophic matrix, is a major site of antimicrobial peptide production (King 1988; Tzou *et al.* 2000) and may also act as a valve, regulating the entry of ingested food into the midgut. Posterior to the cardia, the endodermally derived midgut, with an average length of 6 mm in adult flies, occupies a large part of the abdomen and is commonly regarded as the main digestive/absorptive portion (Demerec 1950; Douglas 2013) (Figure 2, A and B). The Malpighian tubules, tubular excretory organs, discharge at the junction between the midgut and the ectodermally derived hindgut. The hindgut is further subdivided into pylorus (a second valve-like structure), ileum, and rectum, where water/ion exchange may occur (Demerec 1950; Douglas 2013). The muscles surrounding the epithelium are striated, in contrast to the smooth muscles found in mammalian intestines (Sandborn *et al.* 1967). Circular muscles are present throughout the tract, and an outer layer of longitudinal muscles surrounds the midgut. Physiology of the intestine is regulated by autonomic innervation and by hormones (Figure 2C and Figure 5, see *Interorgan signaling* for details of their functions). The gut is further influenced by the tracheal system (Figure 2C), which forms a branched structure surrounding the gut during development (Linneweber *et al.* 2014) and may influence epithelial regeneration in the adult, although the mechanism(s) mediating such interactions remain controversial and provide interesting ground for future work (Guo *et al.* 2013; Z. Li *et al.* 2013).

**Figure 3:**
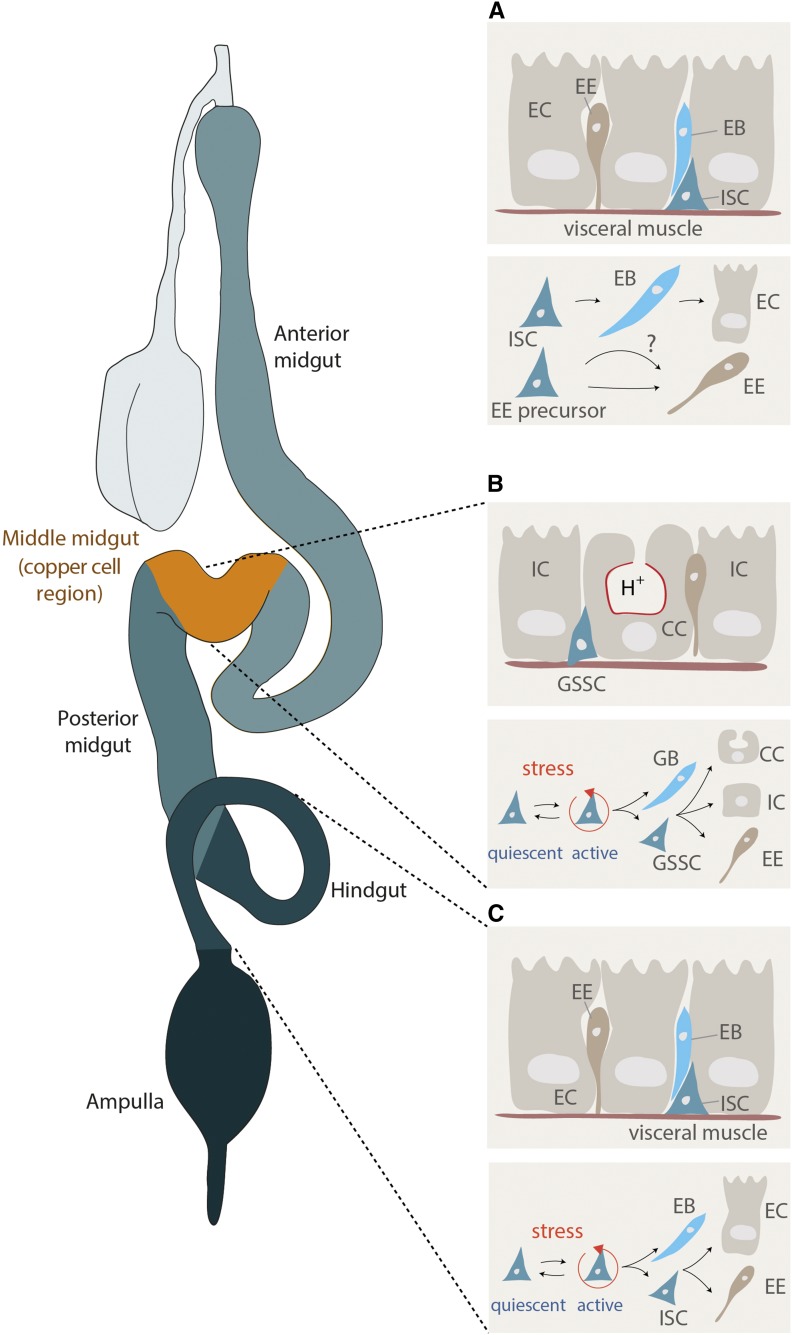
Regional differences in ISC proliferation. (A) General mode of midgut ISC proliferation. See main text for details in *The adult gut and its cell types: genetic and anatomical compartmentalization*. Alternative modes of ISC proliferation - stress induced rather than constitutive - are found in two specific intestinal regions: the copper cell region (B) and the hindgut (C). See main text in *The adult gut and its cell types: genetic and anatomical compartmentalization* for details. CC, copper cell; GB, gastroblast, GSSCs, gastric stem cells; IC, interstitial cell.

Ectodermally derived regions of the intestinal epithelium are relatively poorly understood compared to the midgut, which has been characterized in exquisite detail in recent years. The midgut is grossly subdivided into the anterior midgut, the middle midgut and the posterior midgut, but has been morphologically and molecularly subdivided into 10–14 regions (Murakami *et al.* 1994; Buchon *et al.* 2013b; Marianes and Spradling 2013) (Figure 2B and Figure 3B). Indeed, each midgut region is characterized by specific histological and cellular features (villi size, lumen width), stem cell proliferation rates, physical properties (*e.g.*, luminal pH), and gene expression profiles (Murakami *et al.* 1994; Strand and Micchelli 2011, 2013; Buchon *et al.* 2013b; Marianes and Spradling 2013). The middle midgut (R3) contains a copper cell region in R3ab, which produces gastric acid, followed by a large flat cell region (R3c) with unclear function. Two boundaries flanking this region are inflection points where the midgut folds stereotypically inside the body cavity. Regionalization is not confined to the epithelium—it is also apparent in the muscles, trachea and neurons that surround it (Cognigni *et al.* 2011; Buchon *et al.* 2013b; Marianes and Spradling 2013; Linneweber *et al.* 2014). Although our genetic knowledge of midgut compartmentalization is far from comprehensive, the genes involved in its establishment during development may also play important roles in their adult maintenance. A case in point is the role of the transcription factor Lab, involved in both specification and later maintenance of the copper cell region of the R3 region (Hoppler and Bienz 1994; Buchon *et al.* 2013b; H. Li *et al.* 2013). Graded activities of the Wnt ligand Wingless are observed at several compartment boundaries and may determine their position and identity (Buchon *et al.* 2013b; Tian *et al.* 2016). These boundaries may act as tissue-organizing centers from which Wingless may signal as a morphogen, akin to its roles in development (Buchon *et al.* 2013b; Tian *et al.* 2016). Extensive future studies are needed to gain a detailed understanding of maintenance and plasticity of midgut compartmentalization in the adult.

While significant differences in cellular composition and function exist in the different regions of the adult intestinal epithelium, all regions of the midgut contain ISCs able to regenerate all cell types of their particular region (Buchon and Osman 2015). The ISC lineage was first characterized in the posterior midgut by two groups simultaneously (Micchelli and Perrimon 2006; Ohlstein and Spradling 2006) and is depicted in Figure 3A. Since then, a large number of studies have characterized the regulation of ISCs and their lineages. Midgut ISCs are uniformly interspersed among their differentiated progeny, and are located basally in close proximity to visceral muscles (Figure 2C and Figure 3A). They are, however, heterogeneous in both their cellular behavior and gene expression, which may contribute to specifying compartment differences (Marianes and Spradling 2013; Dutta *et al.* 2015b) (Figure 3, B and C). Consistent with this idea, mosaic analysis has shown that ISCs in a certain region tend to maintain their region’s progeny, and rarely contribute to the production of differentiated cells in adjacent regions.

ISC heterogeneity is established during metamorphosis (Driver and Ohlstein 2014) and is then maintained in cooperation with regional signals from surrounding tissues such as the visceral muscles (see *Stem cells: signals and niches* for details). A multitude of local, paracrine and systemic signals and signaling pathways that control ISC proliferation and differentiation have been identified (see *Stem cells: signals and niches* for details), and changes in ISC function and compartmentalization have been described during tissue damage and aging (Biteau *et al.* 2011; Jiang and Edgar 2011; Buchon *et al.* 2013a,b; Lemaitre and Miguel-Aliaga 2013; Buchon and Osman 2015). ISCs are characterized by the expression of Escargot (Esg) and Delta (Dl), and constitute the majority of cells capable of mitosis in the posterior midgut. ISC maintenance requires the Daughterless protein, as well as transcriptional repression of Notch target genes such as the Enhancer of split complex [E(spl)-C] by a Hairless-Suppressor of Hairless complex (Bardin *et al.* 2010). During regenerative episodes, ISCs in the posterior midgut undergo asymmetric division to give rise to EBs, which retain Esg expression but lose Dl expression while activating Notch signaling. EBs further differentiate into either POU domain protein 1 (Pdm1)-positive absorptive ECs, or Prospero (Pros)-positive secretory EE cells (Micchelli and Perrimon 2006; Ohlstein and Spradlingm 2006, 2007) (Figure 3A). There is evidence for clonal competition during normal homeostasis, whereby loss of ISC through differentiation or death (clonal extinction) may be compensated by increased proliferation/symmetric division of other ISCs (clonal expansion) (de Navascués *et al.* 2012; Kolahgar *et al.* 2015; Suijkerbuijk *et al.* 2016). Tumors may also harness this process to fuel their own growth (Suijkerbuijk *et al.* 2016).

Specification of ECs requires Esg downregulation and activation of Notch and Jak/Stat signaling and the Sox21a and Hindsight transcription factors, while Dpp activity and GATAe contribute to EC production during acute regeneration (Ohlstein and Spradling 2007; Beebe *et al.* 2010; Korzelius *et al.* 2014; Antonello *et al.* 2015 Baechler *et al.* 2015; Zhai *et al.* 2015; Chen *et al.* 2016). Recent studies have refined our understanding of EE specification (Biteau and Jasper 2014; Beehler-Evans and Micchelli 2015; C. Wang *et al.* 2015; Guo and Ohlstein 2015; Zeng and Hou 2015; Sallé *et al.* 2017; He *et al.* 2018). *In vivo* lineage-tracing methods suggest that these cells are generated from precommitted Pros-expressing ISCs, and not, as previously described, as an alternative to EC differentiation from a common EB cell (Figure 3A) (Biteau and Jasper 2014; Guo and Ohlstein 2015; Zeng and Hou 2015). EE specification and differentiation requires less Notch activity than differentiation of EBs into ECs, and involves Phyllopod-mediated repression of the Tramtrack transcriptional repressor, which promotes Scute-mediated activation of Pros (Li *et al.* 2017; Chen *et al.* 2018b; Yin and Xi 2018). Numb and the autophagy protein Atg16 have further been implicated (C. Wang *et al.* 2015; Nagy *et al.* 2017; Sallé *et al.* 2017). EE regeneration from precommitted Pros-positive ISCs may be limited by Slit, an EE-derived ligand for the Roundabout 2 (Robo2) receptor (Biteau and Jasper 2014; Nagy *et al.* 2017). Slit binds Robo2 on ISCs, setting up a negative feedback loop from differentiated EEs that limits further production of these cells (Biteau and Jasper 2014). In this feedback loop, ISCs seem to respond to tissue-wide changes in Slit levels, rather than changes in local concentration, as clonal perturbation of Slit expression or EE concentration is not sufficient to locally influence EE production (Sallé *et al.* 2017). Another study has analyzed EE cell diversity and found that, unexpectedly, Su(H)GBE-positive (Notch active) EBs can give rise to class II EE cells, in addition to ECs (Beehler-Evans and Micchelli 2015). EE differentiation is further promoted by calcium signaling in response to activation of the stretch-activated ion channel Piezo (He *et al.* 2018). While the specification and differentiation pathways regulating EC *vs.* EE lineage specification and differentiation have thus been intensely studied in the past decade, further temporally and spatially resolved lineage tracing studies, potentially coupled with live imaging, will be needed to clarify the exact signaling events governing EE and EC differentiation. Such work is expected to refine the current model describing the defining events promoting EE *vs.* EC lineage specification.

ISCs in the anterior midgut differ in some respects from those in the posterior midgut. For example, proliferation rates and expression of *PAR-domain protein 1* (*Pdp1*) and *Signal-transducer and activator of transcription protein at 92E* (*Stat92E*) reporters are different between anterior and posterior ISCs (Marianes and Spradling 2013), while other transcription factors, such as GATAe, Snail (Sna), and paired-type homeobox transcription factor (Ptx1) have region-specific expression and regulatory roles in ISCs along the digestive tract (Dutta *et al.* 2015b).

Two different ISC populations have been referred to as gastric stem cells. A stem cell pool at the foregut/midgut junction in the cardia can differentiate and migrate to contribute to the crop, the esophagus and the cardia (Singh *et al.* 2011). In the copper cell region of the midgut, which shares some similarity to the stomach in vertebrates, another population of ISCs also referred to as gastric stem cells (Esg- and Dl-positive) generate three different cell types: the acid-secreting copper cells, which express Dve, high levels of Lab, and are detected by an antibody against Cut; interstitial cells, which express Dve and lower levels of Lab; and Pros-expressing EE cells (Strand and Micchelli 2011). Similar to the ISC lineage in the posterior midgut, gastroblasts (the counterpart of the EB in this region) have been identified and proposed to be the precursor cell that generates these three differentiated cell types (Strand and Micchelli 2011) (Figure 3B).

In the hindgut, a ring of ISCs reminiscent of the foregut/midgut junction stem cell pool is found posterior to the pylorus. These hindgut ISCs differentiate into hindgut ECs as they migrate posteriorly (Takashima *et al.* 2008; Fox and Spradling 2009) (Figure 3C). The regenerative properties of the foregut and hindgut have not been extensively investigated, but it is generally assumed that these ectodermal regions of the gut are more quiescent than the endodermal midgut (Fox and Spradling 2009).

## Organ Plasticity

In recent years, the adult midgut has arguably become “the” organ system for the study of adult organ plasticity. We have learned a great deal about the steady-state dynamics of its adult progenitors, as well as their adaptations to challenges, both external (*e.g.*, infection, nutrition) and internal (*e.g.*, aging, reproduction). More recent studies are extending the study of midgut plasticity to nonmitotic cell types, such as the ECs and EE cells of the intestinal epithelium. It is also becoming increasingly recognized that the gut’s anatomical regionalization is associated with striking differences in the turnover and plasticity of different gut regions. This section attempts to provide a comprehensive review of the mechanisms of midgut plasticity. It also discusses their physiological modulation in adult flies, and places the plasticity of the adult midgut in a broader context by briefly contrasting it with the plasticity of other gut regions.

### Stem cells: signals and niches

The activity of ISCs along the digestive tract needs to be specifically and dynamically regulated to adjust tissue turnover to local and tissue-wide needs. Numerous signaling pathways that regulate these processes have been identified. Signaling pathways that influence ISC proliferation and differentiation in *Drosophila* include Notch (Ohlstein and Spradling 2007), Jak/Stat (Jiang *et al.* 2009; Beebe *et al.* 2010; Lin *et al.* 2010), Egfr (Jiang and Edgar 2009; Buchon *et al.* 2010; Biteau and Jasper 2011; Jiang *et al.* 2011), Insulin (Amcheslavsky *et al.* 2009; Biteau *et al.* 2010; Choi *et al.* 2011; O’Brien *et al.* 2011), Jun-N-terminal Kinase (JNK) (Biteau *et al.* 2008), Wg (Lin *et al.* 2008; Lee *et al.* 2009), Target of Rapamycin (Tor) (Amcheslavsky *et al.* 2011; Kapuria *et al.* 2012; Quan *et al.* 2013), Bmp/Dpp (Guo *et al.* 2013; H. Li *et al.* 2013; Z. Li *et al.* 2013; Tian and Jiang 2014; Ayyaz *et al.* 2015), Hippo (Karpowicz *et al.* 2010; Ren *et al.* 2010; Staley and Irvine 2010), Juvenile Hormone (JH) (Reiff *et al.* 2015; Rahman *et al.* 2017), and Ret signaling (Perea *et al.* 2017). ISC proliferation and differentiation also require the Brahma chromatin-remodeling complex (Jin *et al.* 2013). ISC differentiation is further controlled by *esg*-mediated repression of Nubbin (Nub, also known as Pdm1) (Korzelius *et al.* 2014; Loza-Coll *et al.* 2014). The combined action of these signaling pathways influences proliferative activity, self-renewal and differentiation in the ISC lineage in response to a wide range of local and systemic cues. For recent reviews that discuss ISC regulation by these signaling pathways in detail, see Biteau *et al.* (2011), Jiang and Edgar (2011), Buchon *et al.* (2013a), Lemaitre and Miguel-Aliaga (2013), Buchon and Osman (2015), Guo *et al.* (2016), and Gervais and Bardin (2017).

The large number of different signals regulating ISC activity likely results from the need to integrate paracrine, local, systemic, and environmental stimuli to elicit appropriate regenerative responses. During cycles of starvation and refeeding, for example, ISC proliferation is stimulated and switched from an asymmetric mode to a symmetric mode through insulin-like peptide 3 derived from the visceral muscle (O’Brien *et al.* 2011). The visceral muscle also provides Vein and, possibly, Wg ligands that control ISC maintenance and proliferative activity both in homeostasis and during regenerative episodes after epithelial damage (Lin *et al.* 2008; Zhou *et al.* 2013; Biteau and Jasper 2011). The EB, in turn, feeds back to control ISCs proliferation, at least partly by expressing Wg and Unpaired 2 (Upd2) (Cordero *et al.* 2012; Zhai *et al.* 2015; Chen *et al.* 2016). EBs and ECs also limit ISC proliferation through E-cadherin–mediated cell-cell contact (Choi *et al.* 2011; Liang *et al.* 2017). Recent work is starting to provide insight into the integration of these diverse signals. Intracellular calcium signaling, for example, is emerging as a central regulator of ISC proliferation in *Drosophila* in response to a wide range of mitogenic signals (Deng *et al.* 2015; Xu *et al.* 2017).

The diversity of ISC responses to mitogenic signals along the gastrointestinal tract remains poorly understood. ISCs in the posterior midgut and gastric stem cells are regulated by similar signaling pathways, including Wg, Egfr, and Notch (Strand and Micchelli 2011, 2013; C. Wang *et al.* 2014), but differ in their proliferative activity (gastric stem cells are more quiescent than posterior midgut ISCs), and in their response to specific pathways. Loss of Dpp signaling components, for example, causes differentiation defects in gastric-derived lineages, but not in posterior midgut ISCs (H. Li *et al.* 2013). Sustained Dpp expression along the midgut, on the other hand, is sufficient to induce ectopic copper cell formation in the anterior, but not posterior midgut, indicating that additional regional determinants influence stem cell responses to Dpp signaling (H. Li *et al.* 2013). Similarly, activation of Jak/Stat signaling in gastric stem cells leads to their misdifferentiation, generating ectopic EC-like cells in the copper cell region (H. Li *et al.* 2016), while Jak/Stat induces proliferation but does not alter differentiation in ISCs of the posterior midgut (Jiang *et al.* 2009).

### Intestinal plasticity during aging

The digestive tract of adult *Drosophila* has become a powerful model in which to explore aging of barrier epithelia in metazoans (Biteau *et al.* 2011; Lemaitre and Miguel-Aliaga 2013). During aging or after an infection, intestinal compartmentalization is disturbed, as revealed by a strong alteration in gene expression patterns (Buchon *et al.* 2013b). A detailed understanding of the progression of epithelial changes that result in the loss of barrier function in old animals is starting to emerge. In aging flies, ISCs become hyperproliferative, leading to accumulation of misdifferentiated cells that coexpress stem and progenitor cell markers (like Dl and Esg) and differentiation markers (like Notch signaling activity and polyploidy) (Biteau *et al.* 2008; Choi *et al.* 2008; Buchon *et al.* 2009b; Hochmuth *et al.* 2011). An early event causing this is the development of gastric metaplasia, where copper cells transdifferentiate into posterior midgut EC-like (Pdm1-positive) cells, compromising the acidity of the gastric region (H. Li *et al.* 2016). Reduced acidification leads to changes in the compartmentalization and composition of the commensal microbiota, ultimately resulting in commensal dysbiosis and immune deregulation in the midgut epithelium. Dysbiosis, in turn, triggers a secondary inflammatory response, which produces a Dual oxidase (Duox)-induced oxidative burst that damages the epithelium and induces ISC proliferation and misdifferentiation. The resulting epithelial dysplasia, in turn, contributes to the loss of barrier function, which ultimately causes mortality (Rera *et al.* 2012).

Age-related intestinal dysplasia is associated with increased JNK and/or Platelet-derived growth factor (PDGF)/Vascular endothelial growth factor (VEGF) signaling activity (Biteau *et al.* 2008; Choi *et al.* 2008; Buchon *et al.* 2009b; Hochmuth *et al.* 2011), and factors that contribute to dysplasia in the aging intestine include a decline of mitochondrial function in stem and progenitor cells, dysbiosis of gut commensals, inflammatory signals from the fat body, and increased endoplasmic reticulum (ER) stress and Pol III transcriptional activity (Rera *et al.* 2011; Chen *et al.* 2014; Guo *et al.* 2014; Rogers and Rogina 2014; Clark *et al.* 2015; L. Wang *et al.* 2015; Siudeja *et al.* 2015; Filer *et al.* 2017). ISCs of old flies also display frequent somatic mutations, resulting in neoplasia (Siudeja *et al.* 2015). Persistent immune activity has been linked to intestinal hyperplasia and tumor susceptibility (Petkau *et al.* 2017). Neoplasia derived from *Notch*-deficient ISCs has been shown to trigger dysregulation of ISC niche signals, including Egfr ligands and cytokines that activate Jak/Stat signaling, thus contributing to its establishment and development (Patel *et al.* 2015).

The overall longevity of the animal has been shown to correlate with the degree to which these intestinal changes become apparent (Biteau *et al.* 2010), and interventions that specifically target several aspects of intestinal health have been shown to extend the life span of flies reared under laboratory conditions (Rera *et al.* 2011; Ayyaz and Jasper 2013; Chen *et al.* 2014; Guo *et al.* 2014; Clark *et al.* 2015; L. Wang *et al.* 2015).

### Nutritional and metabolic plasticity

The rate of ISC proliferation is substantially but reversibly reduced by long-term nutrient deprivation (McLeod *et al.* 2010; Choi *et al.* 2011; O’Brien *et al.* 2011): an effect recapitulated by genetic manipulations that downregulate/mutate intestinal insulin receptor or downstream pathway components (Amcheslavsky *et al.* 2009; Biteau *et al.* 2010; Choi *et al.* 2011; O’Brien *et al.* 2011). While there is some consensus that nutrient scarcity is, at least partly, relayed to the intestine as a reduction in insulin signaling, different insulin sources and cellular mechanisms have been proposed. Acting in adult intestinal progenitors, insulin signaling promotes ISC proliferation, and is also required to give rise to ECs and EEs (Amcheslavsky *et al.* 2009; Biteau *et al.* 2010; Choi *et al.* 2011; O’Brien *et al.* 2011). Insulin signaling may also control proliferation and differentiation through its actions in EBs, affecting differentiation and ISC/EB adhesion (Choi *et al.* 2011). In contexts of organ resizing (*e.g.*, as the gut grows in the first few days of adult life, or in response to refeeding following prolonged starvation), insulin signaling also supports rapid expansion of the midgut epithelium by promoting symmetric rather than asymmetric ISC divisions (O’Brien *et al.* 2011). The nutrient-driven progenitor expansion and their switch to symmetric divisions are modulated by the two RNA binding proteins Lin-28 and Fmr1, which act antagonistically and post-transcriptionally on the Insulin-like receptor (InR) to modulate how progenitors respond to insulin-like peptide(s) (Chen *et al.* 2015; Luhur *et al.* 2017).

A role for systemic insulin-like peptides in coupling nutrient availability with epithelial turnover has been suggested by ablation of the nutrient-sensitive insulin producing cells of the brain’s *pars intercerebralis* (Amcheslavsky *et al.* 2009; Biteau *et al.* 2010). Other experiments have also revealed a paracrine role for the gut muscle–derived insulin-like peptide Ilp3 in the context of posteclosion and nutrient-driven midgut resizing (O’Brien *et al.* 2011). Ilp3 expression in muscles is nutritionally regulated and sustained by the EE peptide tachykinin (Tk) (Amcheslavsky *et al.* 2014). In this context, it may also be important to consider possible nutritional roles of neuropeptides produced by enteric neurons. Indeed, in larvae, yeast restriction impacts the release of gut neuron-derived insulin-like and Pigment-dispersing factor (Pdf) neuropeptides which, in turn, control the branching of gut terminal tracheal cells (Linneweber *et al.* 2014). The nutritional plasticity of enteric trachea during larval life is physiologically significant, later affecting the ability of adult flies to withstand nutrient scarcity (see *Gut-innervating neurons* for details). A possible contribution of gut trachea and/or neurally derived insulin-like/Pdf neuropeptides to the nutritional modulation of epithelial turnover awaits further investigation. It is also important to underscore that there may be insulin-independent nutritional signals as well as nutrient-independent roles for insulin signaling [see, for example, Quan *et al.* (2013)]. The relative importance of these mechanisms (and, more generally, the effects and relative contribution of nutrition and/or insulin signaling to intestinal homeostasis) are likely to differ depending on the age, microbiota composition, sex, and reproductive status of the experimental flies.

Recent studies are beginning to explore the modulation of epithelial turnover by more direct and/or specific nutritional inputs. A direct action of dietary glutamate acting via metabotropic glutamate (mGluR) receptors in ISC/EBs was suggested by a recent study (Deng *et al.* 2015). ISC proliferation can also be promoted by a lipolysis pathway (Singh *et al.* 2016), or by limiting mitochondrial pyruvate metabolism (Schell *et al.* 2017). Finally, dietary methionine sustains production of the universal methyl donor *S*-adenosylmethionine, which is, in turn, required to sustain ISC proliferation, both directly and through its promotion of Upd3 production in ECs (Obata *et al.* 2018b).

In addition to modulating progenitor dynamics, nutrition also affects the activity of differentiated cells in the intestinal epithelium. For example, young (4n) ECs can undergo a process of ploidy reduction known as amitosis and give rise to new functional ISCs to maintain epithelial integrity following starvation-induced ISC loss (Lucchetta and Ohlstein 2017). An alternative mechanism involving changes in the rate of EC loss has also been proposed (Jin *et al.* 2017). Specific nutrients may also directly change the digestive/absorptive properties of ECs (see, for example, the case of α-amylase in *Digestive enzymes and their regulation*), or the systemic signals produced by EC/EEs (reviewed in *Systemic and EE signals*).

### Sex differences and reproductive plasticity

**Figure 4:**
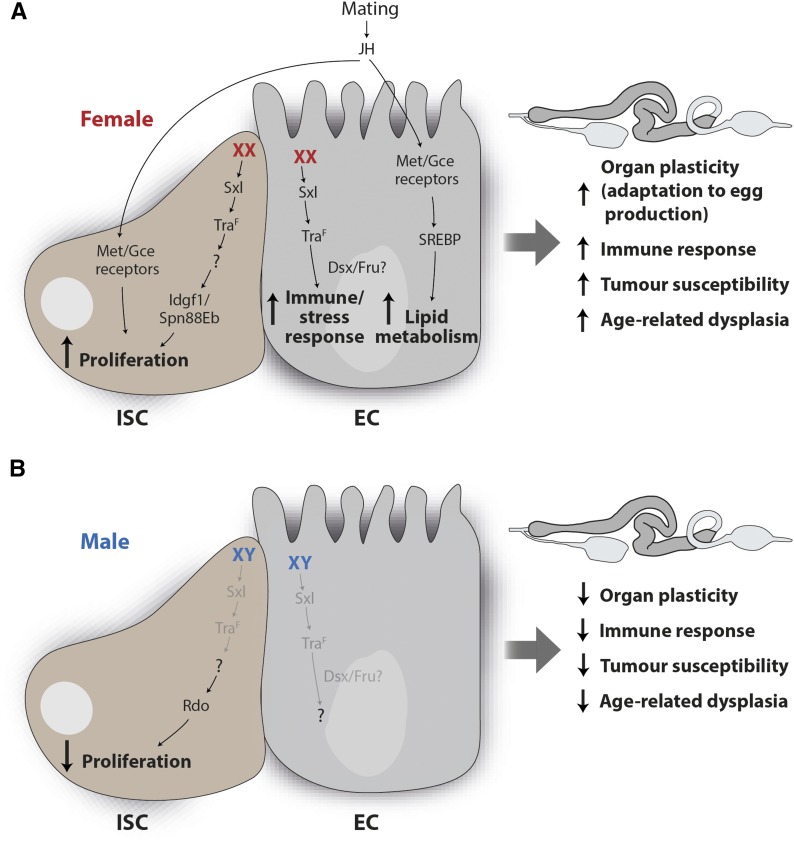
Sex and reproductive differences in ISCs and ECs. (A) Contributions of the intrinsic sex differentiation pathway and the mating-triggered rise in circulating JH to ISC and EC homeostasis in females. (B) Contributions of the intrinsic sex differentiation pathway to ISC and EC homeostasis in males. See section *Sex differences and reproductive plasticity* for details. This figure was inspired by Portman and Biteau (2016).

The nature and significance of sex differences in the *Drosophila* intestine had remained unexplored until recently, despite circumstantial observations pointing to their existence. Indeed, lineage tracing had suggested faster turnover of the adult midgut epithelium in females [data not shown in Jiang *et al.* (2009)], and a method based on quantifying visual features of excreta had revealed modulation of physiological features such as pH and concentration of intestinal contents by sex and reproductive state (Cognigni *et al.* 2011). A more recent study explored sexual dimorphisms more comprehensively in the midgut of adult virgin flies, and revealed extensive sex differences in the expression of genes with putative roles in proliferation, redox, and carbohydrate metabolism (Hudry *et al.* 2016). Adult female ISCs divide more readily than their male counterparts, both in homeostasis and in response to epithelial damage. Increased proliferation in females results from a noncanonical sex differentiation pathway active inside their ISCs. This pathway involves the sex-specific actions of the Transformer (Tra) RNA binding protein downstream of sex chromosome/autosome sensing mechanisms, but is independent of the canonical Tra partner Transformer-2 and the two Tra targets Doublesex and Fruitless (Hudry *et al.* 2016) (Figure 4).

Why are female ISCs more proliferative? A clue was provided by close examination of what happens inside a female fly after mating. In addition to previously described behavioral changes, a single mating dramatically remodels the midgut of a female fly in only 3 days (Reiff *et al.* 2015). Stem cell proliferation, the number of differentiated ECs and the size of the midgut are all increased. Changes in the expression and activity of lipid metabolism regulators in ECs are also apparent. Preventing at least some of these mating-induced intestinal changes reduces egg production, indicating that the reproductive plasticity of the female intestine is important for reproductive success (Reiff *et al.* 2015). While at least one signal upstream of these reproductive changes is a postmating rise in JH (Figure 4), ISCs require their intrinsic female identity to respond to the mating signal(s) by increasing their proliferation (Hudry *et al.* 2016). Hence, the female sexual identity of adult ISCs allows organ resizing for reproductive purposes. The sex difference in midgut size (virgin female midguts are larger and longer than male counterparts, and this dimorphism is further enhanced by mating; Reiff *et al.* 2015; Hudry *et al.* 2016)), also provides an attractive experimental paradigm to explore regulation of organ size. Changes in organ size may not only involve changes in the rate and mode (asymmetric *vs.* symmetric) of stem cell division, but also in the turnover of their progeny; persistent masculinization of adult ISC/EBs in virgin females shrink their gut to a male-like size (Hudry *et al.* 2016). Because virgin male and female guts have comparable ISC density and division mode, the finding that females require a higher proliferation rate to maintain organ size suggests that their EC turnover may also be faster than that of males. Consistent with this idea, genetic manipulations that interfere with EC survival and adhesion in mated females can affect midgut size (Liang *et al.* 2017).

Are there trade-offs to the enhanced plasticity of the female midgut? The female gut is more susceptible to tumorigenic insults, at least partly as a result of the female sexual identity of its intestinal progenitors (Hudry *et al.* 2016) (Figure 4). Another study has begun to shed mechanistic light on the molecular defects contributing to spontaneous, age-related neoplasia and their different prevalence in males and females. The authors also reported a positive correlation between neoplasia and ISC proliferation rate, and further showed that, in males, spontaneous neoplasia often arises through genomic deletions and large structural rearrangements leading to loss of heterozygosity of X-linked tumor suppressors (present in a single copy in males) (Siudeja *et al.* 2015).

As well as neoplasia, other aspects of intestinal (patho)physiology may contribute to differences in life span between the sexes (Figure 4). It has long been known that genetically limiting intestinal proliferation extends the life span of females, but not that of males (Biteau *et al.* 2010). A more recent study reported that age-related dysplasia and intestinal barrier breakdown are both more pronounced in females than males (mated flies were used), and can be ameliorated by dietary restriction: an intervention known to extend life span (Regan *et al.* 2016). Not everything is bad news for female flies; in the same study, male flies were found to be more susceptible to acute intestinal infection and xenobiotic stress. Like the tumors, all these observations could at least partly be explained by the higher proliferation rate of female ISCs, which may help females regenerate their midgut faster after infections, but may also render it more vulnerable to age-related dysplasia.

In light of these recently reported, but apparently extensive sex differences, we encourage the community to control for sex and reproductive state in any future studies.

## Functions

In addition to its obvious roles in nutrient extraction and utilization, the digestive tract responds to the food and bacteria in its lumen to adapt both its own physiology and that of remote organs. In the following sections, we review how the digestive tract senses, digests, and absorbs nutrients, how it interacts with commensal microbes and opportunistic pathogens, and how its different cell populations adapt and signal to the rest of the fly.

### Digestion and absorption

Once food enters the digestive tract, its complex macromolecules are broken down by digestive enzymes before being absorbed by the intestinal epithelium. It is generally accepted that the midgut is the main site of digestion in *Drosophila*, despite evidence for extraoral digestion and enzymatic conversions in the foregut and/or crop of other insects (Lehane and Billingsley 1996). Work primarily in other insects has revealed that digestion can be further modulated by temperature, redox potential, pH, and intestinal transit (Lehane and Billingsley 1996; Douglas 2013). The amount and composition of food available for digestion may also be modulated by gut bacteria (Huang and Douglas 2015). This section describes how nutrients are broken down and absorbed in the adult midgut, as well as the (so far limited) evidence in *Drosophila* for roles of gut acidity and intestinal transit in the context of digestion.

#### Digestive enzymes and their regulation

*Drosophila* feeds on various kinds of decaying plant and fungal material. The relatively complex composition of the material it ingests is paralleled by an impressive array of digestive enzymes dedicated to the handling of carbohydrates, proteins, and lipids; as many as 349, based on bioinformatics predictions, with the largest families corresponding to endo/exo peptidases as well as proteins with carbohydrate or lipase activity (Carlson and Hogness 1985; Ross *et al.* 2003; Horne and Haritos 2008; Horne *et al.* 2009; Tamaki *et al.* 2012; see https://lemaitrelab.epfl.ch/resources for a complete list). Flies may also be able to digest both bacteria and the microbial material found in rotting fruits. Indeed, the presence of 15 different lysozymes in the *Drosophila* genome with no known immune functions suggests that flies may use them to digest peptidoglycan: a major component of bacterial walls (Kylsten *et al.* 1992). Flies also appear to be equipped with chitinases and glucanases that may aid in the digestion of yeasts.

Intriguingly, families of related digestive enzymes are often found as gene clusters in the genome. This applies to Jonah proteases, trypsins, α-esterases, mannosidases, and lipases (Buchon *et al.* 2013b). These gene clusters may have arisen by gene duplication to enhance digestive capacity, and/or to tailor digestive activities to specific portions of the digestive tract following gene duplication and subsequent divergence: a possibility suggested by evolutionary analysis of the α-amylase gene family (Da Lage *et al.* 2000; Zhang *et al.* 2003).

Consistent with regional specialization of digestive functions, the expression of most digestive enzymes is confined to specific segments of the digestive tract (Abraham and Doane 1978; Buchon *et al.* 2013b; Dutta *et al.* 2015a). For example, the expression of some genes coding for enzymes involved in the breakdown of sugars are enriched in anterior (R1/R3) portions of the adult midgut, whereas peptidase genes may be expressed more posteriorly (Dutta *et al.* 2015a). Regional expression of digestive enzymes is also striking in the larva (Harrop *et al.* 2014; Overend *et al.* 2016). However, it is important to consider that the ultimate site of enzymatic activity may not necessarily be equivalent to the site of transcript expression, as enzymes may diffuse in the gut lumen. They may also differ in their positioning and interactions with EC villi or the peritrophic matrix. Indeed, work in other insects has shown that enzymes involved in earlier steps of digestion of macromolecules (*e.g.* α-amylases, proteases) tend to localize to the lumen of the digestive tract, whereas those involved in later steps (maltases, di-peptidases) are more often found in the space between the epithelium and the peritrophic matrix, and are often associated with the surface of gut epithelial cells (Terra *et al.* 1979; Douglas 2013).

The enzymatic activity of the intestine is a key factor determining availability of certain nutrients. It is therefore not surprising that the expression and/or activity of digestive enzymes are tightly regulated in many insects. Modulation by nutrient quality and quantity, neuronal activity, and endocrine signals has been described in insects such as mosquitoes, locusts, cockroaches, or crickets (Wigglesworth 1972; Clissold *et al.* 2010; Douglas 2013), but has not been extensively investigated in *Drosophila*. A substantial reduction of intestinal digestive enzyme activities including trypsin, chymotrypsin, aminopeptidase, and acetate esterase has been reported in flies lacking EE cells, in the absence of obvious effects on food intake (Amcheslavsky *et al.* 2014). The transcription of digestive enzymes with a putative function in breaking down carbohydrate (such as intestinal amylases) is induced by starvation in both larvae and adults (Zinke *et al.* 2002; Chatterjee *et al.* 2014). Their transcription is also sexually dimorphic in adult flies, with many of them showing upregulated expression in males (Hudry *et al.* 2016). It has also long been known that the end products of digestive processes can be repressed by expression of enzymes involved in their production; for example, sucrose and its products, glucose and fructose, have been shown to repress amylase gene expression, an effect known as glucose repression (Hickey and Benkel 1982; Benkel and Hickey 1986; Zinke *et al.* 2002; Chng *et al.* 2014). This reduction in digestive capacity may represent an adaptation to limit dietary sugar absorption during periods of nutritional abundance, given that *Drosophila* is poorly adapted to nutritional excess. Two recent studies have shed light on the repression mechanism. One such mechanism involves the TGF-β/Activin ligand Dawdle (Daw) which, upon refeeding with nutritious sugars (but not non-nutritious sugars) after a period of starvation, reduces the expression of carbohydrate digestive enzymes in the ECs of adult flies (Chng *et al.* 2014). Experiments using whole larvae have also revealed that activation of the intracellular sugar sensor complex Mondo-Bigmax promotes the expression of both *daw* and the transcription factor *sugarbabe* (*sug*) (Mattila *et al.* 2015). *sug* is both necessary and sufficient to repress the expression of amylases. Although further work will be required to clarify how Mondo-Bigmax and TGF-β/Activin signaling intersect, the current data are consistent with a model whereby ECs integrate information about sugar uptake (sensed intrinsically in the intestine by Mondo-Bigmax) and the carbohydrate status of the fat body (relayed by TGF-β/Activin signaling) to modulate expression of the carbohydrate digestive enzymes.

Luminal bacteria can also affect the expression of digestive enzymes, which may, in turn, affect digestive capacity. Indeed, gut-associated bacteria have been shown to modulate the expression of enzymes such as amylases, proteases, and maltases (Erkosar *et al.* 2014). Importantly, the positive effect of microbiota on peptidase gene expression is at least partly responsible for their larval growth-promoting effects in nutrient-poor conditions (Storelli *et al.* 2011; Erkosar *et al.* 2015). Oral bacterial infection is often associated with reduced expression of a broad range of digestive enzymes (*e.g.* lipases, trypsins, amylases) (Buchon *et al.* 2009b; Chakrabarti *et al.* 2012; Erkosar *et al.* 2015; Loudhaief *et al.* 2017; Troha *et al.* 2018), and can lead to hypophagia and changes in excretion (Vallet-Gely *et al.* 2008; Ayres and Schneider 2009; Du *et al.* 2016). The causal links between gut infection, damage, reduced feeding, and expression of digestive enzymes remain to be fully elucidated. Digestive arrest upon infection may be a consequence of gut damage and/or reduced feeding, but might also involve bacteria-to-host signaling. Reduced digestion and some of these other intestinal/feeding changes may represent a host strategy to help limit bacterial ingestion. Alternatively, they may also be a strategy used by pathogenic bacteria to counteract peristalsis and persist in the gut.

In contrast to the relatively abundant data illustrating their dynamic expression, the functions of digestive enzymes remain largely unexplored. Two notable exceptions concern the roles of amylases and a lipase. A functional role for amylases in breaking down complex sugars was revealed by mutants lacking both *amylase p* and *d* (Hickey *et al.* 1988). Unlike wild-type flies, these mutants die on a starch-only diet, but their lethality can be rescued by dietary supplementation with simple sugars: the end products of amylase digestion. Interestingly, mutant lethality can also be bypassed by cohousing the flies lacking amylases with wild-type flies, suggestive of extraoral digestion. Such extraoral digestion may be enabled by regurgitation and/or excretion of amylases. Another functional study concerned the intestinal triacylglyceride (TAG) lipase/cholesterol esterase Magro (Mag). In response to low cholesterol in the diet, expression of the Hr96 nuclear receptor (homologous to the vertebrate LXR receptor involved in regulated cholesterol homeostasis) is upregulated (Bujold *et al.* 2010). Hr96 binds cholesterol and promotes the expression of genes involved in cholesterol homeostasis and lipid breakdown including *mag* (Horner *et al.* 2009; Sieber and Thummel 2009; Bujold *et al.* 2010). Mutant and knockdown experiments are consistent with a dual role for intestinal Mag in breaking down intestinal cholesterol esters to maintain cholesterol homeostasis, as well as enabling TAG breakdown, required for intestinal lipid absorption and peripheral fat accumulation (Sieber and Thummel 2009, 2012). Intestinal *mag* expression can also repressed by a sugar-rich diet in a *foxo*-dependent manner (Karpac *et al.* 2013). Interestingly, this adaptive mechanism becomes chronically active in the aging intestine as a result of JNK pathway activation, disrupting lipid homeostasis and contributing to the age-associated breakdown of metabolic homeostasis (Karpac *et al.* 2013).

#### Absorption of carbohydrates

Following the breakdown of complex carbohydrates by digestive enzymes, a diverse array of transporters internalizes simple sugars into the ECs for further digestion and/or absorption [see Miguel-Aliaga (2012) for a comparative review]. The two major types of glucose transporters known to function in animals, the GLUT/Slc2 family of facilitative glucose transporters and the SGLT/Slc5 family of Na^+^-glucose symporters, have been shown to be expressed and/or active in the intestine of other insects [see, for example, Caccia *et al.* (2005, 2007), Price *et al.* (2007, 2010), Bifano *et al.* (2010)]. One such GLUT-like gene was described in *Drosophila* (Escher and Rasmuson-Lestander 1999), and homologs of other glucose transporters can be found in the *Drosophila* genome. However, their expression and function remain to be investigated. The *Drosophila* genome also harbors a homolog of the more recently characterized SWEET family of sugar transporters (Artero *et al.* 1998; Baker *et al.* 2012).

A disaccharide transporter similar to the transmembrane sugar transporters found in prokaryotes and fungi has also been described in flies [Slc45-1, referred to as Scrt in Meyer *et al.* (2011)]. Slc45-1 belongs to the relatively obscure Slc45 family of transporters, which also includes several human homologs. It is expressed in the embryonic and adult hindgut and can transport sucrose (Meyer *et al.* 2011; Vitavska and Wieczorek 2013). Disaccharide transport may also be achieved by two other trehalose transporters: Tret1-1 and Tret1-2, although the latter shows no trehalose uptake (Kanamori *et al.* 2010).

The possible intestinal activity of these transporters deserves further investigation, not least because of their plastic expression. Indeed, genes with predicted functions in glucose transport are expressed at higher levels in male than female flies (Hudry *et al.* 2016) and, like digestive enzymes, are repressed by a high-glucose diet (Chng *et al.* 2014). It may also be of interest to explore whether putative sweet taste receptors recently shown to be expressed in ECs, enteric neurons, and EE cells (see *Interorgan signaling* for details) are functionally relevant in the context of sugar transport and/or absorption.

#### Absorption of proteins

Proteins are broken down into products of a diverse chemical nature: di- and tri-peptides and a mixture of amino acids. This chemical diversity is paralleled by a broad range of apical and basolateral transport systems, many of which are homologous to known mammalian transporter systems (Boudko 2012; Miguel-Aliaga 2012). These include *Drosophila* homologs of cationic amino acid transporters (Colombani *et al.* 2003), ion-dependent and independent amino acid transporters for neutral amino acids (Martin *et al.* 2000; Goberdhan *et al.* 2005; Miller *et al.* 2008; Reynolds *et al.* 2009) and oligopeptide transporters (Roman *et al.* 1998; Capo *et al.* 2017). Intestinal expression has been reported for the amino acid transporters Pathetic (Goberdhan *et al.* 2005), Minidiscs (Martin *et al.* 2000), NAT1 and other Slc6 family members (Thimgan *et al.* 2006; Miller *et al.* 2008), and the oligopeptide transporters Yin and CG2930, with enriched expression in proventriculus/hindgut and midgut, respectively (Roman *et al.* 1998; Capo *et al.* 2017).

The nature, physiological modulation, and/or significance of many of these amino acid/oligopeptide transporters remains to be investigated. While many of these transport systems may handle dietary nutrients, some may be involved in detection and/or absorption of bacterially derived products. A possible contribution of three *Drosophila* transporters belonging to the Slc15 family of electrogenic (H^+^-coupled) oligopeptide transporters was tested in the context of the *Drosophila* immune response to microbially derived peptidoglycan. A previous study based on expression of ectopic Yin (one of the *Drosophila* Slc15 transporters) in mammalian cells had pointed to potential roles in NF-κB activation downstream of Nod receptors, involved in recognizing peptidoglycan (Charrière *et al.* 2010). However, in flies (which lack Nod-like receptors, and where NF-κB pathway activation by bacteria depends on recognition of peptidoglycan by Peptidoglycan recognition protein (PGRP) family members), neither endogenous mutation of Yin nor that of the two other Slc15 family members CG2930 and CG9444 was found to affect NF-κB activation, peptidoglycan internalization, or its transport from the gut lumen to the circulating hemolymph (Capo *et al.* 2017; Paik *et al.* 2017). One of these two studies found that CG8046, a member of the Slc46 H^+^-driven cotransporter, may instead be involved in the transport of peptidoglycan monomers into the cytosol (Paik *et al.* 2017).

Finally, the broad neutral amino acid transporter NAT1 might also mediate absorption of bacterially derived metabolites (Boudko 2012). NAT1 is expressed in the larval posterior midgut, and is able to transport both L and D isomers of several amino acids (Miller *et al.* 2008). D isomers are particularly abundant in the cell walls of bacteria and can substitute essential L amino acids in the *Drosophila* diet (Geer 1966).

#### Absorption of lipids and sterols

The products of lipid digestion include free fatty acids, glycerol, mono- and diacylglycerols, and phospholipid derivatives. These are absorbed, along with dietary sterols, by intestinal cells. Our knowledge of intestinal lipid transport in *Drosophila* is still rudimentary. Absorption may be at least partly achieved by diffusion of some of these breakdown products across membranes, and may be facilitated by emulsification (Chapman 2013). In contrasts to vertebrates, which emulsify by covering lipids with bile salts, insects achieve emulsification by forming fatty acid-amino acid and glycolipid complexes, as well as fatty acids and lysophospholipid micelles (Chapman 2013). In ECs, the products of lipid breakdown are used to resynthesize diacylglycerols and TAG. These are packaged together with cholesterol and fat body-derived carrier proteins to form lipoprotein particles, which are trafficked throughout the body (Palm *et al.* 2012). This process may help ensure that the products of lipid breakdown are kept at low concentrations inside the ECs, which may facilitate diffusion. Assessment of intestinal lipid accumulation in mutants in which lipoprotein secretion from the fat body is compromised has revealed both anterior and posterior midgut regions as sites of lipid efflux (Palm *et al.* 2012).

In addition to passive diffusion mechanisms, membrane proteins may also contribute to the transport of specific lipid breakdown products. Members of the Cluster of Differentiation 36 (CD36)/Scavenger Receptor Class B type 1 family can mediate the transport of lipoproteins and fatty acids in mammals. Fourteen *Drosophila* homologs of these mammalian genes have been identified (Herboso *et al.* 2011). The intestinal expression of 12 of these *Drosophila* CD36-like genes (Herboso *et al.* 2011) points to their possible function in lipid uptake or handling. Niemann-Pick C1 (Npc1) proteins are 13-transmembrane proteins possessing a sterol-sensing domain that play a key role in intestinal absorption and intracellular trafficking of sterol in mammals (Jia *et al.* 2011). The absorption of sterols is crucial to insects because, unlike mammals, they are unable to synthesize sterols from acetate and thus require a dietary source of sterol for the synthesis of the steroid molting hormone ecdysone. The *Drosophila* genome encodes eight Npc2 and two Npc1 homologs. *Npc1a* and *Npc2a* are broadly required for intracellular sterol trafficking (Huang *et al.* 2007), whereas *Npc1b* is expressed in the midgut and is required for intestinal sterol absorption (Voght *et al.* 2007). Its mutation causes early larval lethality, possibly due to a defect in ecdysone synthesis resulting from sterol deficit. Further double-mutant analyses involving both *Npc1* and *Npc2* family members have, however, pointed to additional, Npc1-independent mechanisms of sterol absorption, possibly involving Npc2 family members (Huang *et al.* 2007; Voght *et al.* 2007). Interestingly, *Npc* genes are targets of the nuclear hormone receptor Hr96, the activity of which is enhanced upon cholesterol scarcity, providing a homeostatic link between dietary cholesterol and its transport machinery (Bujold *et al.* 2010).

The amount of neutral lipid found in the intestine can differ depending on environmental conditions and/or internal state. For example, it accumulates in ECs following changes in the expression of p38 kinase or the Atf3 and Foxo transcription factors (Karpac *et al.* 2013; Chakrabarti *et al.* 2014). Neutral lipid is also increased following depletion of the EE hormone Tk (Song *et al.* 2014), or in sterile female flies after mating (Reiff *et al.* 2015). While accumulation of neutral lipid may result from changes in lipid transport and/or absorption (Song *et al.* 2014; Reiff *et al.* 2015), it may also be reflective of increased intestinal lipogenesis; insect ECs can also make lipids *de novo* from absorbed sugars such as glucose or galactose (Chapman 2013). Consistent with *de novo* intestinal lipogenesis, upregulation/activation of the single *Drosophila* homolog of the mammalian family of sterol regulatory element-binding proteins (SREBPs) and/or some of its targets involved in fatty acid synthesis/activation have been reported in female flies after mating (Reiff *et al.* 2015), in response to skeletal muscle-specific *foxo* manipulations that affect Adipokinetic hormone (Akh) release (Zhao and Karpac 2017), or following Tk depletion (Song *et al.* 2014).

Modulation of intestinal lipid handling appears to be physiologically significant and has been investigated in the context of dietary and reproductive challenges. In addition to the above described diet-dependent regulation of cholesterol levels and peripheral fat stores by HR96 via the TAG lipase/cholesterol esterase Mag (Sieber and Thummel 2009, 2012; Bujold *et al.* 2010), activation of intestinal lipogenesis is key to survival in diet-restricted flies. Indeed, nutrient scarcity induces expression of the sugar sensor transcription factor *sug* in the intestine which, in turn, promotes intestinal lipogenesis. Genetic interference with this response resulted in reduced survival in nutrient-poor conditions (Luis *et al.* 2016). Internal nutritional challenges may be equally dependent on deployment of these intestinal adaptations; for example, to maximize reproductive output in mated female flies (Reiff *et al.* 2015). The signaling mechanism in this case involves a postmating rise in circulating JH which, acting through bHLH-PAS domain proteins Methoprene-tolerant (Met) and Germ cell-expressed (Gce) in ECs, increased SREBP activity and upregulated expression of genes involved in fatty acid synthesis and activation. When the mating-triggered lipid remodeling of ECs was genetically prevented (by means of EC-specific SREBP or JH receptor downregulation), reproductive output was reduced (Reiff *et al.* 2015).

#### Intestinal pH

It is common for many animals to generate localized regions of low pH inside the intestinal lumen. Low luminal pH facilitates protein breakdown, absorption of minerals and metals, and limits the survival of ingested microbes. While mammalian digestion takes place in acidic conditions, insect digestion occurs at neutral or basic pH. *Drosophila* digestion is no exception and large portions of its gut are indeed neutral or mildly alkaline. Luminal pH does, however, display consistent transitions along the length of the intestine and becomes strongly acidic (pH 2–4) in the copper cell region of both larvae and adults (Dubreuil *et al.* 1998; Shanbhag and Tripathi 2009; Overend *et al.* 2016). Posterior to this region, the midgut lumen becomes mildly alkaline again (pH 7–9), but is again acidified in the hindgut (pH 5), partly as a result of discharges from the Malpighian tubules, occurring at the junction between the midgut and hindgut. Final pH adjustments may take place in the rectal ampulla, the acidity of which is strongly affected by diet (Cognigni *et al.* 2011). The cellular and molecular mechanisms involved in establishing and maintaining these pH transitions remain largely unexplored, with one notable exception: the acidic R3 midgut region, where copper cells reside. Copper cells are specialized ECs with a highly invaginated apical membrane, similar to the mammalian gastric parietal cells (Dubreuil 2004). Their role in acid production has been suggested by mutants that interfere with their differentiation, structure, or maintenance. Indeed, acidity is lost in larvae lacking the copper cell–specific homeobox transcription factor Lab, required for copper cell differentiation, and in α*-spectrin* mutants, in which the shape and pattern of copper cells is abnormal (Lee *et al.* 1993; Hoppler and Bienz 1994; Dubreuil *et al.* 1998). In adult flies, genetic interference with copper cell identity or their progressive loss during normal aging are also associated with loss of gut acidity (H. Li *et al.* 2016), with physiological consequences (see *Intestinal plasticity during aging*).

Until recently, the molecular mechanisms of acid secretion had remained puzzling in the absence of an obvious homolog of the H^+^/K^+^-ATPase (the mammalian stomach’s proton pump) in the *Drosophila* genome. Recent experiments have, however, pointed to an involvement of the H^+^ V-ATPase complex. V (vacuolar-type)-ATPases are large multisubunit pumps that transport hydrogen ions in exchange for energy, in the form of ATP. Many of the H^+^ V-ATPase complex subunits are expressed in the intestine, often in a region-specific manner (Allan *et al.* 2005; Buchon *et al.* 2013b; Overend *et al.* 2016), suggesting that the composition, functionality, and/or modulation of the complex may differ in different gut regions. In particular, several complex subunits are enriched in the acidic region of their midgut (Buchon *et al.* 2013b; H. Li *et al.* 2016; Overend *et al.* 2016). Genetic knockdown of the *Vha16-1* gene in adults, which encodes the V_1_ c subunit, or the *Vha100-2* and *Vha100-4* genes in larvae, coding for the V_o_ a subunit, have both revealed their requirement in maintaining the low pH of this region (Lin *et al.* 2015; Overend *et al.* 2016). RNA interference (RNAi) knockdown experiments have also explored the contribution of ion transporters enriched in the acidic region to pH maintenance, and have identified five ion transporters that sustain low pH (Overend *et al.* 2016). These include: the potassium/chloride symporter Kazachoc (Kcc), a member the Slc12 family of electroneutral cation-chloride transporters previously shown to be expressed in several organs including the intestine (Filippov *et al.* 2003; Sun *et al.* 2010); the Slowpoke pore-forming subunit of a calcium-activated K^+^ channel, expressed in neurons, muscles, tracheal cells, and two types of midgut ECs in the copper and iron cell regions (Brenner and Atkinson 1997); the ligand-gated chloride channel pHCL-2 which, in addition to regulating fluid secretion in Malpighian tubules, is expressed in the copper cell, iron, and large flat cell regions of the midgut (Feingold *et al.* 2016; Remnant *et al.* 2016); the carbonic anhydrase CAH1; and the bicarbonate/chloride exchanger CG8177, belonging to the Slc4a1-3 subfamily of anion exchangers expressed in a specific midgut pattern similar to that of pHCl-2 (Dubreuil *et al.* 2010). Of note, a *CG8177* mutant generated in the latter study failed to revel a contribution of this transporter to gut acidity, in contrast to the RNAi knockdown in Overend *et al.* (2016). Differences in the pH indicator dyes used in the two studies may account for this discrepancy. Collectively, these findings suggest that the transport of H^+^, Cl^−^, K^+^, and HCO3^−^ contributes to acid generation in the *Drosophila* midgut.

Recent work is also beginning to shed light on the physiological significance of the luminal pH transitions. Somewhat surprisingly, *lab* mutants lacking copper cells (and, consequently, acid secretion in this region) develop normally, suggesting that maintaining a low pH is not essential for digestion, at least during larval development (Dubreuil *et al.* 2001). More recent studies have revealed links between gut acidity and luminal bacteria. Indeed, preventing acidification of the intestinal lumen in the copper cell region of larvae (achieved by interfering with the V-ATPase complex in copper cells or by downregulating *lab*) is associated with increased bacterial abundance (Overend *et al.* 2016). Preventing acidification by means of *lab* downregulation also increased bacterial abundance in both larvae (Storelli *et al.* 2018) and adults (H. Li *et al.* 2016), and further revealed changes in species composition and their regional localization, with more of them colonizing the posterior midgut (H. Li *et al.* 2016; Storelli *et al.* 2018). Similarly, and as mentioned in *Intestinal plasticity during aging*, the age-dependent decline in copper cell number may contribute to aging from the dysbiosis resulting from acidity loss. The significance of gut acidity during normal development and physiology remains to be elucidated. An intriguing study reported increased adiposity following global knockdown of V-ATPase (Lin *et al.* 2015); an effect that was, however, not apparent when knockdown was confirmed to the midgut in a subsequent study (Overend *et al.* 2016).

#### Absorption of water and osmolytes

To maintain hydration and ionic balance, *Drosophila* flies need to extract water from their diet. This compensates for substantial water loss resulting from metabolic and physiological processes such as respiration. The Malpighian tubules associated with the insect intestine are key to this process, discharging into the junction between the midgut and the hindgut, but there is also a contribution from the intestine itself. In the insect gut, water absorption from the food occurs in the midgut and in the rectum (specifically, in the rectal pads) (Douglas 2013). The rectal pads in the hindgut are also the primary site for reabsorption of ions. The transport of water and ions also plays a key role in the maintenance of ion gradients that sustain active transport in the intestinal epithelium. This is an energetically costly process sustained by ATPases which, like the V-ATPase complex described in the previous section, generate electrochemical gradients that, in turn, drive ion transport through channels, cotransporters, and antiporters.

In insects, ions and water can cross the intestinal epithelium through or between cells (via transcellular and paracellular transport, respectively). Although the relative importance of both mechanisms has not been directly investigated in *Drosophila*, ultrastructural analysis of the larval gut argues against substantial paracellular transport (Shanbhag and Tripathi 2005). The scanning ion-selective electrode technique (SIET) provides a way to probe intestinal gradients for ions such as K^+^, Na^+^, H^+^, or Cl^−^ (Shanbhag and Tripathi 2009; Naikkhwah and O’Donnell 2012). SIET has revealed regional differences in ion concentrations in the larval gut, indicating that K^+^ and Na^+^ absorption occur primarily in the large flat cell and posterior regions of the midgut and, in the case of Na^+^, also in the anterior hindgut (Naikkhwah and O’Donnell 2012). Combined with dietary manipulations, SIET has also uncovered that the mechanisms of intestinal ion transport are plastic; for example, salt stress leads to reductions in K^+^ and Na^+^ absorption and concomitant increases in K^+^ and Na^+^ secretion (Naikkhwah and O’Donnell 2012). The presence or absence of microbiota also seems to have a strong effect on conductance, imparting asymmetry to the epithelium by activating apical membrane conductance (Shanbhag *et al.* 2017). A possible mechanistic link between dietary challenges and transepithelial transport has been provided by the observation that peptide hormones can also modulate *trans*-epithelial ion transport, and they appear to do so in a region-specific manner. Indeed, treatment of larval guts *ex vivo* with Allatostatin A increased K^+^ absorption across the anterior midgut, but reduced it across the copper cells and large flat cells of the middle midgut (Vanderveken and O’Donnell 2014).

The molecular machinery involved in sustaining electrochemical gradients across the intestinal epithelium may be heterogeneous and region-specific, and is only beginning to be characterized. A recent study combining SIET with the use of pharmacological inhibitors has suggested that H^+^ V-ATPase drives H^+^ absorption in the larval caeca and midgut (D’Silva *et al.* 2017). Together with the genetic experiments described in the previous section (Lin *et al.* 2015; Overend *et al.* 2016), this finding lends further support to the idea that the H^+^ V-ATPase complex generates the low luminal pH of the copper cell region. However, this study also showed that there may be other energizing mechanisms involved in establishing intestinal ion gradients, and invoked a Na^+^/K^+^ ATPase that would promote K^+^ secretion in the anterior midgut and the large flat cell zone of the middle midgut: a hypothesis that remains to be genetically tested.

The nature of the channels involved in the transport of ions and water is only beginning to be established. In addition to the Cl^−^, K^+^, and HCO3^−^ channels contributing to the maintenance of acidity in the copper cell region (see previous section), cation/H^+^ antiporters such as the Nhe and Nha channels may, in turn, use the H^+^ electrochemical gradient to achieve *trans*-epithelial transport of other ions (Wieczorek *et al.* 1991; Azuma *et al.* 1995; Chintapalli *et al.* 2015). The activity of the two *Drosophila* Nha members has been explored in some detail. Both are broadly expressed in epithelia including the intestine, and their ubiquitous knockdown decrease survival, especially under salt (specifically Na^+^) stress (Chintapalli *et al.* 2007, 2015; Day *et al.* 2008; Buchon *et al.* 2013b). Salt stress is associated with increased transcription of both channels, intriguingly, in the crop (Chintapalli *et al.* 2015). Experiments in *Xenopus* oocytes indicate that, while Nha2 acts as a canonical Na^+^/H+ exchanger, Nha1 may function as a H+/Cl^−^ cotransporter (Chintapalli *et al.* 2015). In addition to *kcc* (described in the previous section in the context of gut acidity), four other genes encoding homologs of the cation-Cl^−^ Slc12 cotransporters are expressed in osmoregulatory organs (gut, anal pads, and Malpighian tubules) (Filippov *et al.* 2003; Sun *et al.* 2010). Single deletions of two of these genes (*Ncc69* and *CG10413*) are viable, but absence of *Ncc69* does compromise fluid homeostasis in both the Malpighian tubules and the brain (Leiserson *et al.* 2011; Rodan *et al.* 2012). The *Drosophila* genome also encodes seven water aquaporins: small, integral membrane proteins that transport water across cell membranes in response to osmotic gradients created by active solute transport (Verkman 2013). Several aquaporins appear to be expressed in specific intestinal regions (Kaufmann *et al.* 2005; Chintapalli *et al.* 2007; Buchon *et al.* 2013b), but their role in maintaining fluid balance remains to be established.

As we acquire better understanding of the mechanisms involved in shuttling ions and water across intestinal membranes, it will also be important to gain deeper insight into the functional significance of these processes. In addition to their contributions to maintaining gut pH gradients and water/ion balance (described above), recent studies are also pointing to roles for ion gradients in metal absorption (see next section) and cold tolerance (Andersen *et al.* 2017; Overgaard and MacMillan 2017).

#### Absorption of metal ions

Metal ions such as iron, copper, and zinc are essential micronutrients required for the correct folding and/or activity of a broad range of enzymes. The biological actions of metal ions such as iron and copper often rely on the fact that they can exist as ions of multiple valences. This redox activity can contribute to oxidative stress and therefore requires sophisticated mechanisms to avoid metal toxicity by controlling their availability. The contribution of the intestine to metal homeostasis has not been extensively investigated, but several studies have identified important (and, often, evolutionary conserved) molecular mediators of uptake, storage and export. Early studies pointed to two specific midgut regions, the so-called copper cell and iron regions, as the most likely sites of metal ion absorption. The copper cell region becomes bright luminescent orange upon copper ingestion due to the fixation of copper by metallothionein (Poulson and Bowen 1952; McNulty *et al.* 2001), and appears to be an important site of accumulation of ingested radioactively labeled copper (Poulson *et al.* 1952) [although see Lauverjat *et al.* (1989)]. Similarly, the iron cell region in R4a is stained by Prussian blue (a histological stain commonly used to visualize the presence of iron) and also accumulates exogenously administered radioactive iron (Poulson and Bowen 1952; Missirlis *et al.* 2007). Subsequent studies have explored the molecular machinery involved in the intestinal uptake, intracellular trafficking, and efflux of metal ions. These studies, described in some detail below, have confirmed roles for the copper/ion regions, but have also revealed that the mediators of metal handling are more broadly distributed along the midgut than previously thought.

##### Copper

The uptake of copper may be mediated by three Copper transporter 1 family importers: Ctr1A, B, and C. None of these transporters are uniquely expressed in the copper cell region (Chintapalli *et al.* 2007; Buchon *et al.* 2013b; Marianes and Spradling 2013; Overend *et al.* 2016). Ctr1C expression may be confined to the male gonad. Ctr1A is broadly expressed and leads to developmental arrest when mutated (Turski and Thiele 2007; Hua *et al.* 2010). Based on its expression in EC membranes (Selvaraj *et al.* 2005), Ctr1B may be devoted to intestinal copper transport. It is expressed during larval stages, and is up-regulated in response to low dietary copper (Zhou *et al.* 2003). It is also the only copper importer identified as a direct target of the metal-responsive enzyme MTF-1 (Southon *et al.* 2004; Selvaraj *et al.* 2005). *Ctr1B* mutants die as larvae when copper is scarce, while heterozygotes are viable but show pigmentation defects, reflecting the requirement for copper as a cofactor for enzymes involved in pigmentation such as the tyrosinase enzyme (Zhou *et al.* 2003). In addition to Ctr1 family members, another protein has been proposed to contribute to copper transport: Malvolio (Mvl), the *Drosophila* homolog of the divalent transporter ion transporter 1 (DMT1). Mvl appears to be primarily involved in the transport of dietary iron in the gut (see *Iron* section below), but its expression levels can modulate copper content in both S2 cells and in the gut (Southon *et al.* 2008). Flies lacking *Mvl* are viable, but are sensitive to dietary copper excess.

Copper export from the intestine to the hemolymph is mediated by the P-type ATPase ATP7, which is localized at the basal membrane (Burke *et al.* 2008). ATP7 expression is both copper- and MTF-1-dependent (Burke *et al.* 2008; Mercer *et al.* 2017). Unlike its mammalian counterparts, however, it may not translocate from the Golgi to the plasma membrane in response to copper (Burke *et al.* 2008; Mercer *et al.* 2017). In addition to the transporters of copper into and out of cells, a third group of proteins known as copper chaperones is known to play a key role in copper homeostasis in mammalian cells (Palumaa 2013; Navarro and Schneuwly 2017). Copper chaperones ensure the safe handling and specific delivery of potentially harmful copper ions to a variety of essential copper proteins. However, their intestinal roles remain to be investigated.

##### Iron

Iron functions as a cofactor in nearly 100 enzymes. Earlier findings defined the midgut iron cell region as the site of Prussian blue stain accumulation (Poulson and Bowen 1952), showed that iron was present in the form of ferritin (Locke and Leung 1984), and established that excess dietary iron resulted in its active excretion from cells in the copper cell region into the intestinal lumen (Poulson and Bowen 1952). Since then, studies of iron regulation in flies have identified molecular mediators of iron import, export, and storage. These studies have also revealed that, in contrast to what we know so far about the transport of other metals, several of the key proteins involved in iron homeostasis in mammals, such as EPO or hepcidin, are not found in flies. Different anatomy and physiological requirements may account for these molecular differences; for example, the different way in which oxygen is distributed across the body and, probably as a consequence, the fact that *Drosophila* and other insects lack oxygen-carrying blood cells (Mandilaras *et al.* 2013).

Iron uptake into ECs is, at least in part, mediated by Mvl, the *Drosophila* homolog of the DMT1 that mediates iron import in mammals. *Mvl* is expressed in anterior and posterior parts of the midgut as well as in Malpighian tubules, brain, and testis (Folwell *et al.* 2006), and its absence results in depleted iron stores and reduced intestinal iron accumulation (Bettedi *et al.* 2011). Dietary iron export from ECs into the hemolymph requires an intracellular iron transfer step mediated by iron transporter Zip99C (also known as Zip13): an ER/Golgi-resident protein previously presumed to function as a zinc importer (Xiao *et al.* 2014). Zip99C knockdown results in whole-body (but not midgut) iron deficiency. Zip99C may be required for ferric iron loading into the ferritin storage complex, which titrates free iron and is highly expressed in the midgut, particularly in iron cells (Mehta *et al.* 2009; Xiao *et al.* 2014). Unlike mammals, *Drosophila* ferritin chains contain secretion signals and may be secreted into the hemolymph (Nichol *et al.* 2002). Consistent with a role for ferritin in iron export from ECs, midgut-specific ferritin knockdown results in intestinal iron accumulation and systemic iron deficiency (Tang and Zhou 2013).

Multicopper oxidase (Mco) proteins may also play a role in dietary iron absorption. *Drosophila* has four Mco proteins that might function as ferroxidases (Lang *et al.* 2012; Wang *et al.* 2018) [although see Peng *et al.* (2015)]. Knockdown of *Mco1*, located on the basal surfaces of the intestine and Malpighian tubules, results in decreased iron accumulation in both midguts and whole insects (Lang *et al.* 2012). A role for Mco3 in intestinal iron absorption has also been suggested by the observation that *Mco3* mutants accumulate iron in the iron region of the intestine, and restore the depleted iron stores of *Mvl* mutants (Bettedi *et al.* 2011). Its effects on overall iron homeostasis seem, however, milder than those of *Mco1* mutants (Bettedi *et al.* 2011; Lang *et al.* 2012; Wang *et al.* 2018). The fate of absorbed iron once it has reached the hemolymph remains to be investigated, as does the mechanism involved in its trafficking. Transferrins serve as iron transport carriers between cells in mammals, and the *Drosophila* homologs may play similar roles in flies (Mandilaras *et al.* 2013). Alternatively (or additionally), transferrins may also play developmental roles in the intestine, such as that described for the transferrin-2 septate junction assembly (Tiklová *et al.* 2010).

##### Zinc

Zinc is a redox-neutral element that plays key structural, catalytic, or coactivator roles in enzymes, as well as acting as a cofactor in the zinc finger domain of hundreds of transcription factors. Several transporters have been identified that mediate zinc uptake and efflux. Generally, zinc uptake into cells is mediated by ZIP transporters, whereas its efflux from into the hemolymph is mediated by ZnT transporters. The *Drosophila* genome encodes 10 Zip genes (Richards and Burke 2016) and seven ZnT genes, although some of these proteins may mediate intracellular zinc transport (Lye *et al.* 2012; Richards and Burke 2016; Xiao and Zhou 2016). The zinc transport activity of several of these ZIP/ZnT proteins has often been indirectly inferred from homology to their mammalian counterparts, or from the effect of their overexpression/knockdown (alone or in combination) in the adult fly eye, where developmental defects in zinc handling can lead to a rough eye phenotype. A few studies have attempted to characterize the endogenous transporters mediating intestinal zinc transport in larvae. These studies are consistent with a partially redundant role for Zip42C.1 and Zip42C.2 (expressed on the apical side of a subset of ECs) in uptake from the lumen into the EC (Wang *et al.* 2009; Qin *et al.* 2013). Larvae lacking another Zip, *Zip89B*, are resistant to high dietary zinc and upregulate expression of *Zip42C.1* and *Zip42C.2*, suggesting that Zip89B may also contribute to zinc uptake (Richards *et al.* 2015). Zip88E was also once thought to contribute to the dietary absorption of zinc (Dechen *et al.* 2015). However, the recent generation of a *Zip88E-Gal4* reporter is suggestive of expression in subsets of neurons and EE cells, pointing to possible roles in metal sensing or transport these cell subsets (Richards *et al.* 2017).

Exit of zinc from the ECs into the circulation appears to be primarily mediated by zinc exporters ZnT63C and ZnT77C (Wang *et al.* 2009; Qin *et al.* 2013), and may not require handling by intracellular organelles (Qin *et al.* 2013; Richards and Burke 2015). *ZnT63C* is expressed at the basal membrane of a subset of ECs in an unspecified midgut region, as well as in Malpighian tubules. Downregulation of *ZnT63C* results in developmental arrest when dietary zinc is scarce, while its overexpression causes hypersensitivity to zinc (Wang *et al.* 2009). Strikingly, the human homolog of *ZnT63C* (human *ZnT1*) can rescue the sensitivity of *ZnT63C* knockdown flies to dietary zinc restriction (Wang *et al.* 2009). The expression and contribution of ZnT77C are less well characterized, and a possible role in manganese rather than zinc transport has been suggested (Richards and Burke 2016).

While these studies have begun to shed light on the relative importance of these transporters in the intestine, more work will be needed to confirm their metal specificity, establish the contribution of specific intestinal regions to zinc handling, and clarify where in the cells these proteins function. Functional analyses of zinc homeostasis at the cell and tissue levels will be facilitated by methods to visualize and quantify zinc with cellular or subcellular resolution. Changes in intracellular zinc levels have been inferred from activation of the zinc-activating reporter *MtnB-eYFP* (Lye *et al.* 2012), but recent approaches have also made use of synchrotron X-ray fluorescence microscopy (M. W. Jones *et al.* 2015) or zinc-sensitive dyes such as Fluozin-3 (Filipiak *et al.* 2012; Groth *et al.* 2013; Tejeda-Guzmán *et al.* 2018). Functional studies of zinc homeostasis may also benefit from the recent transcriptional characterization of zinc detoxification responses in *Drosophila* cells (Mohr *et al.* 2018).

Of note, the *white* mutant strain commonly used as an experimental control strain lacks zinc storage granules (Tejeda-Guzmán *et al.* 2018). Investigations of zinc homeostasis or zinc-related phenotypes may consequently require the use of wild-type flies, or flies containing mini-White transgenes.

##### Maintaining metal homeostasis

The expression of many genes involved in metal handling is dynamically regulated by dietary metal availability. This includes the above-mentioned Ctr1B and ZIP/ZnT transporters, but also the five known *Drosophila* metallothioneins (MtnA, MtnB, MtnC, MtnD, and MtnE) (Egli *et al.* 2006a; Atanesyan *et al.* 2011). The expression of Mtns (cysteine-rich proteins able to sequester metals) is sensitive to copper, cadmium, and zinc load and, in the intestine, both their endogenous expression and induction by metals are highly regionalized (Durliat *et al.* 1995; Egli *et al.* 2006a,b; Atanesyan *et al.* 2011). Consistent with a role for Mtns in metal detoxification, flies lacking all four of them are sensitive to copper, cadmium, and zinc load (Egli *et al.* 2006b). Similarly, the expression of the conserved copper-binding zinc finger transcription factor MTF-1 is also dynamically regulated. Strikingly, MTF-1 appears to be able to promote the expression of different target genes depending on whether a specific metal is scarce or too abundant (Selvaraj *et al.* 2005; Chen *et al.* 2008; Sims *et al.* 2012): the copper transporter Ctr1B when copper is scarce (Zhang *et al.* 2001), and the Mtn proteins in intestines exposed to high-copper diets (Egli *et al.* 2006b; Balamurugan *et al.* 2007; Norgate *et al.* 2007). Relatively little is known about how the iron absorption machinery is physiologically regulated. In mammalian cells, iron-response proteins (IRPs) repress the translation of proteins such as ferritin while increasing expression of DMT1 and Transferrin in response to low dietary iron (Mandilaras *et al.* 2013). Two IRP1 homologs have been described in flies (Rothenberger *et al.* 1990; Muckenthaler *et al.* 1998; Lind *et al.* 2006) that might play similar roles (Mandilaras *et al.* 2013).

As well as regulating transport and sequestering metal excess in proteins such as Mtns, maintaining metal homeostasis may also require active regulation of metal storage. For example, larvae have been shown to accumulate copper following a period of copper scarcity (Balamurugan *et al.* 2007). The subcellular localization of the different components of the uptake/efflux machinery may also be tightly regulated. A recent study illustrated the importance of localizing transporters correctly, and began to shed light on the mechanisms involved. In this study, midgut-specific knockdown of the vacuolar-type H^+^ ATPase subunit *VhaPPA1-2* or the aquaporin homolog *big brain* resulted in mislocalization of apical copper and zinc uptake proteins, as well as whole-body pigmentation phenotypes consistent with copper deficiency (J. Wang *et al.* 2014).

Possible interactions between the molecular machinery involved in handling different metals also deserve further investigation; for example, the finding that elevated levels of dietary zinc lead to increased ferritin protein in the posterior midgut points to possible links between intestinal zinc and ion homeostasis (Gutiérrez *et al.* 2010). Similarly, high levels of dietary copper have long been known to reduce midgut iron accumulation (Poulson and Bowen 1952) and whole-body iron stores (Bettedi *et al.* 2011). The mechanism involved remains to be established but might involve inhibition of iron-sulfur cluster biosynthesis by copper (Vallières *et al.* 2017; Marelja *et al.* 2018). Finally, behavioral adaptations (*e.g.* avoidance of high copper food) may also contribute to maintaining metal homeostasis (Balamurugan *et al.* 2007).

#### Transit and excretion

Adjusting the repertoire of digestive enzymes and nutrient transporters is only one of several mechanisms by which digestion and absorption can be coupled to internal or environmental challenges. There is now evidence that both the transit of food along the alimentary canal as well as its subsequent excretion are also subject to active modulation, which may affect nutrient extraction and/or utilization.

In larvae, excretion appears to be stereotypical and cyclic; sequential contractions of the posterior hindgut and anal sphincter have been described, leading to opening of the anal slit to expel feces out of the lumen every 38 sec at 25° (Zhang *et al.* 2014). In the adult, pulse-chase experiments using food dyes have revealed that food can travel the entire length of the digestive tract in less than 1 hr (Wong *et al.* 2008). However, this process can be regulated at the levels of intestinal capacity, transit, and excretion by multiple external and internal factors. For example, the amount of food retained in the crop is much larger in starved then refed flies than in flies fed *ad libitum* (Edgecomb *et al.* 1994; Wong *et al.* 2008), and starvation also reduces defecation rate long before the gut is emptied (Cognigni *et al.* 2011). Chronic food deprivation in larval life has been shown to subsequently increase excretion in adult flies, even in nutritional conditions or genetic backgrounds in which no differences in food ingestion were observed (Urquhart-Cronish and Sokolowski 2014). One mechanism by which nutrient availability may be coupled with intestinal transit and excretion may involve the Diuretic hormone 44 (Dh44) brain-derived neuropeptide (described in *Gut-innervating neurons*). Several other peptides have also been shown to affect peristalsis of intestinal muscles (see *Systemic and EE signals* for details). However, the relevant source of these peptides (brain *vs.* EE cells) remain to be investigated, as does their physiological regulation.

Internal challenges such as reproduction or infection can also affect transit and excretion. For example, female flies actively engaged in reproduction reduce their defecation rate despite increasing their food intake (Cognigni *et al.* 2011). By contrast, ingestion of food-borne pathogens such as *Erwinia carotovora* increases defecation rate, promoting bacteria expulsion (Du *et al.* 2016). In the latter case, the mechanism involved requires the TrpA1 channel in EE cells. Bacteria-derived uracil leads to Duox-dependent production of highly reactive HOCl, which activates the TrpA1 channel in EE cells. Loss of TrpA1 results in increase bacterial persistence and can exacerbate the mortality rate of immunocompromised flies (Du *et al.* 2016).

In addition to adapting transit time and excretion frequency, flies can also change the nature of their excreta. For example, a recent study has pointed to the existence of a diet-dependent factor in excreta, only produced following ingestion of nutritive sugars (Abu *et al.* 2018). This factor may convey the presence of food to other flies, causing them to aggregate around it. The pH of excreta is also physiologically regulated in response to both external (nutritional) and internal (reproductive) challenges. The hindgut may contribute to the pH adjustment, which may help offset the excess acid produced by these physiological stressors (Cognigni *et al.* 2011). The water content of excreta can also be actively modulated: excreta become more concentrated in response to metformin treatment (Slack *et al.* 2012) or, more physiologically, during reproduction. Indeed, as well as reducing defecation rate, mated females flies also retain fluid, yielding fewer, more concentrated excreta (Cognigni *et al.* 2011). Changes in intestinal fluid retention are likely to involve the distal part of the hindgut (namely, the rectum and/or rectal glands), given its known role in water reabsorption in other insects (Douglas 2013), and may help maximize absorption at a time of high nutritional demand. Mechanistically, the fluid retention observed in mated females does not result from passive allocation of fluid/nutrients to egg production, or from changes in renal function. Instead, it is at least partly mediated by the sex peptide transferred by males during copulation (Cognigni *et al.* 2011; Apger-McGlaughon and Wolfner 2013), which ultimately affects the HGN1 subset of hindgut-innervating neurons (Cognigni *et al.* 2011; see *Gut-innervating neurons* for details).

In future, it may be important to clarify the connections between intestinal fluid retention, absorption, peristalsis, and excretion. The diverticulated crop may well prove to be a key organ in this regard, given that its differential peristalsis and engorgement can determine whether food is temporarily stored or released into the midgut for digestion and absorption (Stoffolano and Haselton 2013). Intriguingly, mutations in the *drop dead* gene affect the nervous system but are also associated with increased crop size, reduced transfer of ingested food from the crop to the midgut, and reduced defecation (Buchanan and Benzer 1993; Peller *et al.* 2009). A link between muscle peristalsis and epithelial absorption has been suggested by characterization of mutants lacking the key regulator of energy homeostasis AMPK (Bland *et al.* 2010). *AMPK*α mutants are developmentally delayed but, strikingly, this phenotype can be rescued by visceral-muscle specific reintroduction of AMPKα which, by promoting muscle peristalsis, enhances nutrient intake and supports growth of the whole animal. It will also be important to consider possible effects of transit and excretion on microbiota, recently shown to modulate the energy of their host through their consumption of dietary nutrients (Huang and Douglas 2015). More generally, it will also be interesting to explore how differences in intestinal transit and excretion affect as yet unrelated aspects of intestinal homeostasis, such as stem cell renewal, immunity, and senescence.

### Gut microbiota and immunity

#### Intestinal microbiota

##### Drosophila and its microbial communities

Our understanding of the nature and impact of *Drosophila* microbial communities has increased dramatically in the past decade. *Drosophila* live and feed on an ephemeral ecological niche, rotting fruit, where it is constantly exposed to yeasts and bacteria (Broderick and Lemaitre 2012). Unlike mammals or social insects (Engel and Moran 2013), *Drosophila* do not harbor a core microbiota of defined bacterial species like mammals or social insects. However, *Drosophila*-associated microbes still have a profound effect on its physiology. The associations between *Drosophila* and its microbiota can be qualified as “open,” as there is connection between the microbial communities inside the gut and in the external environment; external microbes colonize the host, and gut microbes are released in the external environment as part of excreta. Released bacteria can, in turn, modify the ecological niche in a way that favors colonization by bacteria that are beneficial to the fly (Winans *et al.* 2017; Storelli *et al.* 2018). Contrasting with the general view that the gut microbiota of *Drosophila* is unstable, two stably associated bacterial strains have been recently reported; such stability would favor their spread into the environment (Pais *et al.* 2018). Both the stability and subsequent dynamics of this association have been shown to differ between bacteria from the wild or from laboratory flies (Obadia *et al.* 2017). Studies comparing wild-caught and laboratory fly stocks have shown that the gut of *D. melanogaster* is an environment with low bacterial diversity (1–30 species), and that the most commonly found species are members of three major families: *Lactobacillaceae* (*e.g.* *Lactobacillus*, *Leuconostoc*), *Acetobacteraceae* (*e.g.* *Acetobacter*, *Gluconobacter*) and, sometimes, *Enterobacteriaceae*. Yeasts such as *Hanseniaspora* or *Saccharomyces* are also found (Chandler *et al.* 2011; Wong *et al.* 2011; Broderick and Lemaitre 2012). Interestingly, flies tend to favor microbiota diversity, and are attracted to specific bacteria-yeast compositions able to provide specific metabolites, such as derivatives of ethanol and acetate catabolism (Fischer *et al.* 2017). Different bacterial strain differ in their spatial colonization of the gut, which may help maintain diversity (Obadia *et al.* 2017).

The *Drosophila* microbiota found in the laboratory is less diverse than that found in the wild, and can be shaped by food composition. For instance, a food medium enriched in sucrose should favor the predominance of *Acetobacter* that are efficient at processing it (Huang and Douglas 2015), and starvation decreases bacterial loads and affects interfly variation differentially depending on whether bacteria are wild or laboratory isolates (Obadia *et al.* 2017). The composition of the fly’s microbiota is further influenced by the host genotype (Wong *et al.* 2013; Dobson *et al.* 2015; Early *et al.* 2017). Thus, bacteria found in the gut are therefore a subset of those found in the food substratum. Larvae, which are continuous feeders, have a more constant and abundant microbiota (Storelli *et al.* 2011). In the laboratory, emerging adults have almost no bacteria in their gut, and frequent flipping of flies on sterile medium tends to reduce the microbiota load of adults (Blum *et al.* 2013; Wong *et al.* 2013; Broderick *et al.* 2014). Many studies have shown that microbial loads increase upon aging (see *Intestinal plasticity during aging* for details), with a shift in bacterial composition (Buchon *et al.* 2009b; Clark *et al.* 2015)

##### The effect of microbiota on host traits

Germ-free flies can be easily cultivated in the laboratory, but the presence of live bacteria becomes crucial when flies are raised on a poor diet. The importance of bacteria as a food source is suggested by the large repertoire of fly enzymes that digest bacterial cell wall (see *Digestive enzymes and their regulation*). The microbiota also complements *Drosophila* metabolism by providing vitamins (notably, thiamine), cholesterol from yeast, and by enhancing survival on ethanol or high-glucose diets (Whon *et al.* 2017; Sannino *et al.* 2018). Microbiota is not just a food source: it needs to be alive to mediate its beneficial effects. Analysis of germ-free and gnotobiotic flies has unveiled key roles of bacteria in promoting larval growth and oogenesis (Shin *et al.* 2011; Storelli *et al.* 2011; Ridley *et al.* 2012; Elgart *et al.* 2016). *Drosophila*-associated bacteria can simulate digestion and generate metabolites that impact insulin or TOR growth pathways (Shin *et al.* 2011; Storelli *et al.* 2011). Many laboratory experiments, however, do not allow us to distinguish whether these effects result from the direct action of bacteria on the gut, or from their processing of the food medium the flies feed on, which could facilitate its digestion. The presence of indigenous bacteria also maintains a basal level of ISC activity by stimulating Nox and Duox reactive oxygen species (ROS) signaling in the gut (R. M. Jones *et al.* 2015). At the same time, it promotes low-level antibacterial immunity which, in turn, regulate microbiota loads together with ROS and acidity (Buchon *et al.* 2009a; Broderick *et al.* 2014). Interestingly, the Immune deficiency (Imd) immune pathway that mediates most antibacterial response in the gut also regulates the expression of a subset of microbiota-regulated digestive enzymes (Erkosar *et al.* 2014, 2015; Matos *et al.* 2017). There may be trade-offs to the microbiota’s broadly beneficial effects; the high levels of ROS and AMPs produced by old flies to control microbiota stress the epithelium. This increases epithelial cell turnover, contributing to a shift from epithelium renewal to dysplasia, which eventually compromises gut function (Buchon *et al.* 2009a; Guo *et al.* 2014). As discussed above, one of the initial triggers for the age-dependent microbiota expansion may be the loss of acidity resulting from loss of copper cell identity (see *Intestinal plasticity during aging* for details). Many contradictory findings involving (or not) the microbiota may be accounted for by the specific diets used in different studies. Thus, the role of the microbiota can only be established as an interaction with the nutritional environment. For instance, *Drosophila* bacteria can promote the lifespan of flies raised on a poor diet by complementing the food (Yamada *et al.* 2015). By contrast, germ-free flies are generally longer-lived if raised on a rich diet. Microbiota composition can shape food choice and egg laying behaviors by buffering adult flies from the lack of dietary essential amino acids (Leitão-Gonçalves *et al.* 2017). Intriguingly, the *Drosophila* microbiota can influence mating preference (Sharon *et al.* 2010), although this finding was not reproduced by other laboratories (Leftwich *et al.* 2017). Changes in microbiota may also exert the long-term effects of early-life stress; indeed, transient exposure to low concentrations of oxidants was found to extend adult lifespan by selectively depleting microbiota of an *Acetobacter* species (Obata *et al.* 2018a).

In conclusion, there is increasing evidence for strong effects of gut-associated microbes on a broad range of host traits. Such effects may result, at least partly, from the different ways in which microbes modulate *Drosophila* nutrition. More broadly, the *Drosophila* microbiota studies have provided a new perspective on host-microbe interactions by shedding light on the plasticity, flexibility and mutual benefits of such interactions, from the microbial as well as the host side.

#### Mucosal immunity

The digestive tract of *Drosophila* is a major entry route for infectious agents such as viruses, bacteria, fungi, and parasites. It also contains transient bacteria such as plant pathogens that use the fruit fly as a vehicle for transmission. Some of these bacteria express virulence factors that promote gut colonization (Basset *et al.* 2003). Several constitutive or inducible layers of defense help the fly protect against ingested pathogens. To provide protection against abrasive food particles and enteric pathogens, the intestinal epithelium is entirely lined by a chitinous matrix (Hegedus *et al.* 2009). While the foregut and hindgut are lined by an impermeable cuticle, a more permeable structure — the peritrophic matrix — protects the midgut. The peritrophic matrix is composed of chitin fibrils and chitin-binding proteins that are assembled in the proventriculus, remodeled along the midgut and eventually degraded at the mid-hindgut junction (Kenmoku *et al.* 2016). The presence of a peritrophic matrix and epithelial tight septate junctions explains why only rare pathogens are capable of breaching the gut and penetrate the hemolymph compartment (Nehme *et al.* 2007; Kuraishi *et al.* 2011; Bonnay *et al.* 2013; Shibata *et al.* 2015). Most entomopathogenic bacteria, such as *Bacillus thuringiensis*, *Pseudomonas entomophila*, or *Serratia marcescens*, harm their host while in the lumen by producing pore-forming toxins, which cross the peritrophic matrix and target the midgut epithelium (Opota *et al.* 2011; Lee *et al.* 2016). Some bacteria such as *P. entomophila* also secrete proteases that degrade the peritrophic matrix, thus facilitating the action of pore-forming toxins (Shibata *et al.* 2015). Peritrophic matrix components are cross-linked by enzymes such as transglutaminase. There is a trade-off between high cross-linking to allow resistance to the action of pore forming toxins and low cross-linking to increase permeability for nutrient absorption (Kuraishi *et al.* 2011; Shibata *et al.* 2015). Interestingly, intestinal epithelia exposed to hemolysin, a pore-forming toxin secreted by *Serratia marcescens*, undergo an evolutionarily conserved process of thinning followed by recovery of their initial thickness within a few hours. In this process, ECs extrude most of their apical cytoplasm, including organelles, yet do not lyse. Epithelial thinning may allow fast and efficient recovery to intestinal infections, with pore-forming toxins acting as alarm signals (Lee *et al.* 2016). Ingestion of pathogenic bacteria also induces a transient blockage of food uptake: a behavioral response that limits further microbial contamination (Chakrabarti *et al.* 2012; Keita *et al.* 2017). As described in *Intestinal pH*, the acidity of the copper cell region contributes to the elimination of ingested bacteria. Harsh conditions in the crop, protease activities, lysozymes, and peristalsis are all thought to contribute to host defense, but their precise roles have not yet been investigated.

In addition to these constitutive host defense mechanisms, local production of antimicrobial peptides and ROS provides two complementary and inducible defense mechanisms in the gut. Ingestion of Gram-negative bacteria triggers the expression of several antimicrobial peptide genes in specific domains along the digestive tract (Buchon *et al.* 2009b). This response is regulated by the Imd pathway upon recognition of peptidoglycan by either a transmembrane recognition receptor PGRP-LC in the ectodermal parts of the gut or an intracellular receptor PGRP-LE in the midgut (Bosco-Drayon *et al.* 2012; Neyen *et al.* 2012). Intracellular sensing by PGRP-LE likely involves the translocation of peptidoglycan monomers from the gut lumen into ECs. The gut antibacterial response is kept in check by several negative regulators of the Imd pathway, notably enzymatic PGRPs that scavenge peptidoglycan (Lhocine *et al.* 2008; Paredes *et al.* 2011; Charroux *et al.* 2018). Excessive and deleterious immune activation is observed in flies lacking these negative regulators (Paredes *et al.* 2011). Peptidoglycan fragments can also cross the gut and remotely induce the production of antimicrobial peptides by the fat body (Neyen *et al.* 2012; Charroux *et al.* 2018), and can interfere with neuronal octopamine release to reduce egg laying (Kurz *et al.* 2017). Together with JNK signaling, the Imd pathway also promotes EC delamination; cell shedding is likely to contribute to bacterial elimination (Loudhaief *et al.* 2017; Zhai *et al.* 2018). Both Toll and melanization pathways are functional in the foregut and hindgut but not in the midgut (Buchon *et al.* 2013b). The Toll pathway contributes to resistance to oral infection by *Drosophila* C virus, but the protection mechanism, whether physiological or immune, is not yet known (Ferreira *et al.* 2014). Transcriptionally, transcription factors such as Nub coordinate the response to bacterial infection (Lindberg *et al.* 2018). The second inducible host defense mechanism is the production of ROS by Duox or the NADPH oxidase Nox (Ha *et al.* 2005; Jones *et al.* 2013). Duox is activated upon the sensing of uracil released by pathogenic bacteria (Lee *et al.* 2013). ROS are not only bactericidal, but increase defecation of food-borne pathogens by activating the HOCl receptor TrpA1 in EE cells (Du *et al.* 2016). A side effect of ROS production is damage to the intestinal barrier. Efficient and rapid recovery from a bacterial infection is possible only when the immune response is coordinated with epithelial renewal to repair the damage caused by infection (Amcheslavsky *et al.* 2009; Buchon *et al.* 2009a; Jiang and Edgar 2009). While the regulation of Duox has been extensively studied (Lee *et al.* 2018), the precise contribution of both Duox and Nox in immunity and epithelium renewal remains to be elucidated. High epithelial turnover is triggered in response to infection by the release of secreted ligands of the Upd family, which activate the Jak/Stat pathway in progenitors. The Jak/Stat pathway further regulates a subset of putative antifungal peptides with homology to Drosomycin (Osman *et al.* 2012). Epithelial renewal has also been shown to function against oral viral infection (Sansone *et al.* 2015). While the midgut has been the focus of most studies, the crop and the proventriculus are both expected to play a role in the defense against pathogens. Systemic infections have an effect on the gut, which results in increased epithelial renewal since circulating hemocytes can remotely activate ISC proliferation by providing a source of Upd (Takeishi *et al.* 2013; Chakrabarti *et al.* 2016; Guillou *et al.* 2016). Upd3 expression is induced in hemocytes by JNK signaling after septic injury, and hemocyte-derived Upd2 and Upd3 can activate ISC proliferation (Chakrabarti *et al.* 2016). The gut thus turns out to be a critical organ to survival by systemic infection (Takeishi *et al.* 2013; Chtarbanova *et al.* 2014; Chakrabarti *et al.* 2016). Upon oral infection, hemocytes are further recruited to the midgut and release Dpp, which can trigger ISC proliferation in the early phase of the injury response but, as a consequence of changes in Dpp receptor composition, also restrain continuous proliferation in the recovery phase (Ayyaz *et al.* 2015). The sources and regulation of both Upds and Dpp in these responses are complex, with ligands being produced by hemocytes, epithelial cells, and even the visceral muscle in a dynamic fashion during the regenerative response (Osman *et al.* 2012; Guo *et al.* 2013; Tian and Jiang 2014, 2017; Ayyaz *et al.* 2015; Chakrabarti *et al.* 2016; Jiang *et al.* 2016; Houtz *et al.* 2017; Tian *et al.* 2017). Recent work has started to explore the role of specific signaling pathways and transcription factors in the regulation of Upd3 expression in the gut, and has found key roles for the transcription factors Scalloped (Sd), Mothers against dpp (Mad), and D-Fos. Accordingly, the Hippo, TGF-β/Dpp, Src, and p38-dependent MAPK pathways were found to regulate Upd3 in ECs. It can be anticipated that such studies will provide the basis for a more detailed exploration of the dynamics of Upd expression, both during regeneration and in normal homeostasis (Houtz *et al.* 2017; Zhai *et al.* 2018). The role of hemocytes in oral infection models remains to be further investigated as a recent study failed to observe a defect in ISC proliferation following *Ecc15* oral infection in flies in which hemocytes were ablated through the expression of BAX (Chakrabarti *et al.* 2016). While further work is thus needed to elucidate the relative and temporal contribution of specific signals and cell types to the infection-triggered increase in epithelial turnover, it is increasingly apparent that intestinal epithelial renewal is sensitive to insults originating both from the lumen and the hemocoel.

### Interorgan signaling

Throughout this review, we have encountered multiple examples of coordination between different intestinal functions such as digestion, absorption, transit, and excretion. We have also summarized evidence for coupling between intestinal physiology and the functions of other organs; for example, the effects that several intestinal manipulations can have on food intake. Such coordination and coupling rely on both intercellular and interorgan communication. Enteric neurons and endocrine signals are emerging as important mediators of these processes.

#### Gut-innervating neurons

##### Anatomy of enteric innervation

**Figure 5:**
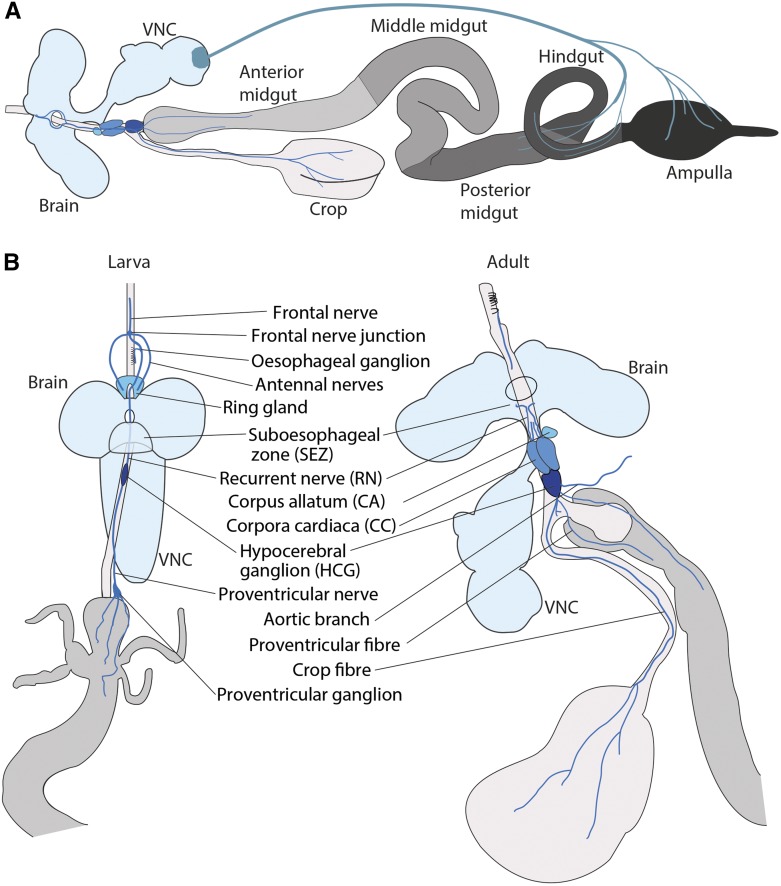
Innervation of the adult intestine. (A) Enteric innervation of the adult digestive tract. Neurons located in the brain and enteric ganglia contribute to the innervation of anterior portions (see B and Gut-innervating neurons section for details). Central neurons with cell bodies in the posterior segments of the abdominal ganglion of the ventral nerve cord (VNC) send axons in the hindgut nerves toward the pylorus, which extend anterior along the posterior portion of the midgut. The hindgut nerves also branch to innervate the rectal ampulla and rectum. (B) Stomatogastric nervous system ganglia and nerves contribute to enteric innervation of the larval esophagus and anterior midgut (left) and adult esophagus, crop, and anterior midgut (right). In adults, the larval proventricular and hypocerebral ganglia have fused. The fused hypocerebral ganglion is adjacent to the *corpus allatum* and *corpora cardiaca*, which have moved posteriorly from their larval head position to the main body of the adult fly.

The adult digestive tract receives innervation from three distinct sources: the stomatogastric nervous system (Hartenstein *et al.* 1994; Gonzalez-Gaitan and Jackle 1995; Pankratz and Hoch 1995; Spiess *et al.* 2008); the *corpora cardiaca*, neurosecretory structures which, in adult flies, are fused with one of the stomatogastric ganglia (the hypocerebral ganglion) (Lee and Park 2004); and neurons located in the central nervous system (CNS), which extend their axons toward three different portions of the digestive tract (Miguel-Aliaga and Thor 2004; Miguel-Aliaga *et al.* 2008; Cognigni *et al.* 2011; Talsma *et al.* 2012; Schoofs *et al.* 2014b) (Figure 5, A and B). The Ret receptor tyrosine kinase, crucial for the development of the mammalian enteric nervous system, has recently been shown to contribute to the development of stomatogastric ganglia in flies (Myers *et al.* 2018). Ret remains expressed in most, if not all, gut-innervating neurons in adults, including stomatogastric as well as CNS neurons (Perea *et al.* 2017). The anatomy of different stomatogastric ganglia and their interconnectivity are summarized in Figure 5B for both larvae and adults.

In contrast to mammalian gastrointestinal tracts, profusely innervated throughout their entire length, innervation of the fly’s digestive tract is confined to three distinct portions: its anterior-most portion comprising the pharynx, esophagus, crop and anterior midgut; the midgut/hindgut junction; and the posterior hindgut (Hartenstein *et al.* 1994; Spiess *et al.* 2008; Cognigni *et al.* 2011; Schoofs *et al.* 2014b) (Figure 5, A and B). Muscle valves are present in all three regions, consistent with the idea that peristaltic regulation and intestinal transit are important functions of gut-innervating neurons. Most neurites terminate on the visceral muscles, but some appear to reach the underlying epithelium, particularly in the esophagus, proventriculus, pyloric valve, and rectal ampulla (all of which are ectodermally derived epithelia) (Cognigni *et al.* 2011; Kenmoku *et al.* 2016). This is suggestive of neuronal regulation of epithelial properties such as secretion or absorption (see next section for two examples). Not all innervation is efferent; taste neuron afferents from the pharynx are known to send their axons to the suboesophageal zone (the taste center of the fly brain), where they target a distinct domain adjacent to the projections of other (leg/labellum) gustatory receptor neurons (Stocker 1994; Wang *et al.* 2004; Marella *et al.* 2006; Ito *et al.* 2014). Posterior to the pharynx, sensory innervation is relatively scarce. Dendrites emanating from peripheral sensory neurons are apparent in the anterior and posterior-most regions of the digestive tract (Cognigni *et al.* 2011), and appear most abundant in the esophagus and anterior midgut (P. Cognigni and I. Miguel-Aliaga, unpublished data). Few peripheral cell bodies are apparent on, or close to the intestine (Cognigni *et al.* 2011; P. Cognigni and I. Miguel-Aliaga, unpublished data), and many of them are likely to be efferent neurons residing in the stomatogastric ganglia. An anal sensory neuron controlling defecation in larvae has been investigated in some detail (Zhang *et al.* 2014; see below for functional relevance).

Enteric innervation is chemically diverse. In the anterior portion of the larval and/or adult midgut, neurites positive for serotonin and various neuropeptides have been described, including Akh, Dh44, Myosuppressin, and possibly Allatostatin C and FMRFamide (or an FMRFamide-like peptide such as the NPY-like neuropeptide short neuropeptide F [sNPF]) (Budnik *et al.* 1989; McCormick and Nichols 1993; Nichols *et al.* 2002; Lee and Park 2004; Schoofs *et al.* 2014b; Dus *et al.* 2015). On the larval and/or hindgut, neurites positive for Pdf, Ion transport peptide, and Proctolin are found (Anderson *et al.* 1988; Nässel *et al.* 1993; Miguel-Aliaga and Thor 2004; Dircksen *et al.* 2008; Dircksen 2009). Notably, all three innervated regions receive insulinergic innervation from the CNS; the *pars intercerebralis* insulin-producing cells extend axons beyond the ring gland that innervate the anterior midgut and crop in adult flies, and the insulin-like peptide 7 (Ilp7)-producing neurons of the abdominal ganglion innervate the midgut/hindgut junction and the rectal ampulla (Cao and Brown 2001; Miguel-Aliaga *et al.* 2008). Intriguingly, putative dendritic termini of both kinds of insulin-producing neurons are found in very close proximity in the CNS, suggesting that the release of different insulins to the different portions of the digestive tract may be coregulated centrally (Cognigni *et al.* 2011; see following section).

##### Intestinal and nonintestinal roles of enteric neurons

Functional studies of insect innervation have predominantly concerned the control of peristalsis and peptide hormone secretion. The neuronal mechanisms that generate and propagate the peristaltic waves that allow transit and digestion are not well understood, but recent studies have suggested that the central pattern generators that generate feeding rhythms in larvae are located in the suboesophageal zone (Hückesfeld *et al.* 2015). Clusters of serotonergic neurons located in this area innervate the anterior portions of the digestive tract including stomatogastric ganglia, and play an important role in modulating the frequency of food ingestion and esophageal peristalsis (Schoofs *et al.* 2014b, 2018). Once initiated, peristaltic waves may propagate by myogenic transmission, given the lack of innervation of some portions of the digestive tract, including a significant portion of the midgut (Cognigni *et al.* 2011). There is precedent for myogenic transmission of electrical activity in the absence of innervation across the gastroduodenal junction of cats, dogs, and primates (Bortoff and Davis 1968). Studies of peristaltic regulation in *Drosophila* have primarily concerned the effects of neuropeptides such as Allatostatins, Myosuppressin, or Drosulfakinins on *ex vivo* intestinal preparations (Kaminski *et al.* 2002; Price *et al.* 2002; Palmer *et al.* 2007; Vanderveken and O’Donnell 2014). They have also ascribed distinct roles for these peptides in the modulation of crop or anterior midgut contractions in adults. Two studies have combined these classical contraction assays with more modern genetic and imaging techniques to demonstrate both intestinal and nonintestinal roles in the regulation of muscle peristalsis for a set of hindgut-innervating neurons located in the abdominal ganglion of the CNS: the Pdf-expressing neurons (Talsma *et al.* 2012; Zhang *et al.* 2014). It was found that this neural source of Pdf (a neuropeptide related to mammalian vasoactive intestinal polypeptides, better known for its roles in the central circadian clock) promotes peristalsis of hindgut muscles and sustains the defecation cycle in larvae (Zhang *et al.* 2014). Pdf can also promote contractions of the muscles of the ureters, the proximal part of the Malpighian tubules (Talsma *et al.* 2012). Hence, some enteric neurons may use the digestive tract as a docking site to exert their functions on other internal organs at some distance.

Less is known about the physiological modulation of peristalsis and the roles of neurons in this context. A link between nutrient availability and peristalsis has been provided by the finding that six neurosecretory cells in the *pars intercerebralis* of the adult brain secrete Dh44 in response to nutritious sugars (Dus *et al.* 2015). At least some of these neurons innervate the anterior midgut and crop, and gain- and loss-of-function experiments targeting Dh44 or its receptor(s) revealed a role for Dh44 in promoting intestinal motility and excretion (Dus *et al.* 2015). Both Dh44 neurons and the gut-innervating insulin-producing neurons of the *pars intercerebralis* are innervated by Hugin-producing neurons that suppress food intake and induce locomotion, providing a possible link between food-related behaviors and intestinal physiology (Schoofs *et al.* 2014a).

Beyond peristalsis, recent studies have revealed epithelial roles for gut-innervating neurons. Their role in the control of fluid balance have been revealed by a method based on the semiautomated analysis of defecation behavior in adult flies, providing quantitative readouts for food intake, fluid/ion balance, and intestinal transit (Cognigni *et al.* 2011; Wayland *et al.* 2014). The HGN1 neurons, a subset of 2-5 CNS neurons located in the posterior segments of the abdominal ganglion, innervate the hindgut and the rectum, with a subset of their neurites projecting through the visceral muscles to reach the underlying epithelium (Cognigni *et al.* 2011). Silencing of HGN1 neurons resulted in increased defecation rate, and further revealed that, perhaps as a result of their epithelial innervation, HGN1 neurons are required for the postmating changes in intestinal fluid retention (described in *Transit and excretion*). A more recent study of larval innervation has confirmed a contribution of both HGN1 neurons and the Pdf hindgut-innervating neurons to the defecation cycle. It has further revealed that these two neuronal populations are activated sequentially, and has established their direct action on the hindgut and/or anal sphincter muscles (Zhang *et al.* 2014). Intriguingly, this study has further revealed that these neurons receive presynaptic input from anal sensory neurons that may sense anal opening via the NompC TRP channel. It will be interesting to explore whether the anal sensory neurons are part of the neuronal circuit that relays the sex-peptide driven changes that ultimately affect the function of HGN1 neurons, possibly providing a neuronal link between intestinal fluid retention and the reduced defecation observed in mated females. A role for gut-innervating neurons in the maintenance of epithelial turnover has also been suggested by the finding of anatomical proximity between enteric neurites in the posterior midgut and adult somatic intestinal progenitors, and the reduced ISC to EC differentiation resulting from downregulating Hedgehog (Hh) signaling (albeit pan-neuronally) (Han *et al.* 2015). The more anterior innervation of the proventriculus (the gut portion where peritrophic matrix is made) may also play a role in maintaining gut permeability. This was inferred from the finding that inactivation of a relatively broad subset of neurons, including a subset of anterior midgut-innervating neurons results in an abnormal proventricular structure, increased permeability of the epithelial barrier, and increased susceptibility to oral bacterial infection: all suggestive of defects in the production of peritrophic matrix (Kenmoku *et al.* 2016).

Finally, functional analyses of the two different sets of gut-innervating, insulin-producing neurons has revealed that, in addition to the intestinal muscles and epithelium, intestinal trachea are an important cellular target of their actions (Linneweber *et al.* 2014). During larval life, the branching of the tracheal terminal cells is plastic and highly dependent on dietary yeast. Such plasticity is controlled by different insulin- and/or Pdf-expressing gut-innervating neurons, which act via insulin and Pdf receptors in tracheal cells. *In vivo* calcium imaging of larval Ilp7 neurons has suggested that these neuropeptides are released in response to nutrients or hypoxia to promote tracheal branching, providing a neuronal link between nutrients and tracheal plasticity. Such plasticity during larval life is important in the context of adaptive responses to malnutrition in later life; flies with reduced gut tracheation readily mobilize their lipid stores and survive better when faced with nutrient scarcity. The mechanism by which reduced gut tracheal branching results in these adaptations remains to be established. In adult flies, genetic inactivation of insulin-producing neurons resulted in opposing effects on the hyperphagic response triggered by nutrient scarcity; Kir2.1-mediated silencing of the insulin-producing cells of the brain *pars intercerebralis* that innervate the anterior midgut reduced this response, whereas silencing of the hindgut-innervating Ilp7 neurons increased it, and also resulted in higher circulating glucose (Cognigni *et al.* 2011; Olds and Xu 2014). It remains to be established, however, whether these effects result from the effects of the insulins on trachea, their systemic actions, or their effects on other intestinal cell populations.

Little is known about the significance of the sparse sensory innervation of the intestine. One notable exception at its anterior-most end are the pharyngeal taste neurons. *pox-neuro* (*poxn*) mutant flies lacking taste sensory function in the legs and labial palps retain expression of sweet taste receptors in their pharynx and a preference for sweet compounds, suggesting a pharyngeal contribution to sugar detection (Galindo and Smith 2001; LeDue *et al.* 2015). Further insight into the taste circuit relaying this pharyngeal sensory signal was provided by the identification of a subset of interneurons (the so-called IN1 neurons) receiving input from the pharyngeal sensory neurons. The activity of IN1 neurons is exquisitely dependent on the amount and duration of feeding (Yapici *et al.* 2016). Posterior to the pharynx, in the gastrointestinal tract, the contribution of sensory innervation to nutritional homeostasis remains to be investigated. Postingestive sensory feedback from the gut has been assumed to inhibit feeding based on work in other insects; for example, severing the recurrent nerve or the medial abdominal nerve, which transmit information from the gut to the brain, results in overconsumption in blowflies (Dethier and Gelperin 1967). Recent work in flies lends support to this idea; whereas severing the medial abdominal nerve did not affect food consumption, severing the recurrent nerve elevated consumption of sucrose but not water or bitter solutions (Pool *et al.* 2014). The existence of neuronal stretch receptors on the gut that monitor the volume of ingested food is, to some degree, supported by both neurophysiological and anatomical data in several other insects (Dethier and Gelperin 1967; Belzer 1978; Chapman 2013; Stoffolano and Haselton 2013). However, the existence and molecular nature of these receptors in *Drosophila* remains to be established. Intriguingly, six peripheral neurons on the proventriculus have been shown to express the gustatory receptor Gr43a, which is also expressed by some pharyngeal neurons and can function as a fructose receptor in central neurons (Miyamoto *et al.* 2012; Mishra *et al.* 2013; Miyamoto and Amrein 2014). These proventricular neurons extend dendritic processes into the foregut lumen, and a subset of their axons innervate the midgut, whereas another subset extend along the esophagus, forming a nerve bundle with axons of gustatory receptor neurons projecting toward the suboesophageal ganglion. Hence, they may relay nutritional information back to central/more anterior neurons or act locally on the gut. Establishing their roles will require genetic tools able to target the enteric subset without affecting the central or peripheral Gr43a-expressing neurons.

### Systemic and enteroendocrine signals

As well as using neurons, the *Drosophila* intestine can also communicate with other organs through systemic signals. Intestinal physiology is modulated by both extrinsic hormonal signals (emanating from endocrine glands, neuroendocrine structures, or organs such as the fat body) as well as by its own peptide hormones, produced by EE cells. In turn, gut-derived signals such as EE cell-derived peptide hormones can have long-range effects on other internal organs.

#### 

##### Enteroendocrine hormones

EE cells are relatively abundant in flies, accounting for 5–10% of midgut epithelial cells compared to 0.4–0.6% in the mammalian small intestine (Cheng and Leblond 1974; Micchelli and Perrimon 2006; Beehler-Evans and Micchelli 2015). At least 95% of them express peptide hormones, often more than one and with regional stereotypy (Veenstra *et al.* 2008; Veenstra 2009; Reiher *et al.* 2011; Beehler-Evans and Micchelli 2015). The developmental program of EE cells shares similarities with that of neurons, probably reflecting a common phylogenetic origin (Hartenstein 2006; Hartenstein *et al.* 2010, 2017). Consistent with this idea, all known EE peptide hormones (possibly with the exception of one of the insect CCHamides; S. Li *et al.* 2013) are also produced by the brain. Acting through these hormones, EE cells may play “neural-like” roles in regulating intestinal physiology and/or conveying intestinal/nutritional state to other cell types or organs—roles that may be particularly prominent in the midgut given the relatively sparse innervation of this gut region. Flies lacking all EE cells (*scute* mutants) are viable, relatively normal in terms of food intake and fertility, but are shorter-lived and, as described below, display abnormal intestinal homeostasis (Amcheslavsky *et al.* 2014).

There is some evidence for local paracrine actions of EE peptides. A role for EE cells on muscle peristalsis was suggested by the finding that ablation of Diuretic hormone 31 (Dh31)-expressing EE cells or Dh31 downregulation both reduced muscle peristalsis of muscles in the larval anterior midgut, which may function as a valve to minimize mixing of acidified and nonacidified food in the acidic region of the midgut (LaJeunesse *et al.* 2010). Two links between EE cells and ISC proliferation have also been described, both involving a visceral muscle relay (Amcheslavsky *et al.* 2014; Scopelliti *et al.* 2014). Adult EE cells produce Bursicon which, by signaling through the Bursicon/DLGR2 receptor in visceral muscle, represses the production of the visceral muscle-derived mitogen Vein and, consequently, ISC proliferation. The net effect of EE cells on epithelial turnover may, however, be the opposite: an independent study found that EE cell depletion in *scute* mutants compromised the nutrient-dependent midgut growth that occurs posteclosion (Amcheslavsky *et al.* 2014). This effect was partly accounted for by the lack of EE cell-derived Tk, which normally promotes expression of the visceral muscle-derived Ilp3 insulin-like peptide shown to sustain ISC proliferation and nutrient-dependent midgut growth (O’Brien *et al.* 2011; Amcheslavsky *et al.* 2014). Somewhat paradoxically, another study found that Tk production in EE cells was increased during starvation, and that EE cell-derived Tk acts on its receptors in ECs to suppress SREBP-mediated intestinal lipogenesis, contributing to the loss of systemic lipid storage (Song *et al.* 2014). Finally, a recent comparative fly–mouse–human study has pointed to neurotensin-like signaling from EE cells to ECs in flies, with effects on lipid metabolism and AMPK activation. Indeed, expression of mouse neurotensin from *Drosophila* EE cells (and possibly also peripheral sensory neurons) promoted lipid accumulation in both standard and high-fat diets in the midgut, fat body, and oenocytes, and also decreased gut AMPK activation (J. Li *et al.* 2016). The effect was dependent on expression of the Pyrokinin 1 receptor in ECs, but did not seem to be mediated by EE cell-derived Pyrokinin 1, pointing to an involvement of a different ligand (J. Li *et al.* 2016).

Recent work has also provided evidence for systemic roles for EE-derived peptide hormones. A high-sugar diet leads to increased midgut EE cell number and enhanced production of EE-derived Activin ligand (in this case, Activin-β rather than Daw) (Song *et al.* 2017). Mirroring the activin-mediated fat-to-gut signaling involved in sucrose repression (described in *Digestive enzymes and their regulation*), midgut-derived Activin-β binds to the TGF-β receptor Baboon in fat cells which, in turn, leads to enhancement of Akh signaling it the fat body and consequent hyperglycemia (Song *et al.* 2017). There may also be a contribution of EE CCHamides to feeding, growth rate, sensory perception, and olfactory behavior (Farhan *et al.* 2013; Ren *et al.* 2015; Sano *et al.* 2015). CCHamides are recently discovered insect hormones (Roller *et al.* 2008; Hansen *et al.* 2011). Their expression is promoted by nutrient availability and sites of expression include the gut EE cells, a subset of central neurons and, possibly, the fat body (S. Li *et al.* 2013; Ren *et al.* 2015; Sano *et al.* 2015). Their receptors are expressed in the nervous system including the insulin-producing neurons, and are absent from the gut (S. Li *et al.* 2013; Sano *et al.* 2015). Although this expression is suggestive of gut to brain (or peripheral nervous system) signaling, definitive proof that EE cell-derived hormones can reach central neurons awaits further investigation. Although not strictly gut-derived, a new peptide hormone produced not by EE cells, but by an adjacent secretory gland may have provided the most compelling example to date of gut-to-brain communication. Indeed, Limostatin (Lst) peptide is produced by the *corpus cardiacum*: the Akh-producing gland which, in the adult, is found adjacent to the hypocerebral ganglion on the gastrointestinal tract, at the junction between the esophagus and anterior midgut. Lst is released in response to nutrient restriction and suppresses insulin production by the insulin-producing cells of the brain *pars intercerebralis*. *Lst* mutant flies accumulate excess fat and display phenotypes associated with insulin excess (Alfa *et al.* 2015).

Relatively little is known about the physiological modulation of EE peptide hormone release. Experiments using the cytoplasmic calcium reporter CaLexA—normally used to visualize neuronal activation—have suggested that a subset of Dh31- and Tk-expressing EE cells are activated by dietary protein and amino acids (Park *et al.* 2016). At least 12 gustatory receptors have been recently reported to be expressed in subsets of EE cells (Park and Kwon 2011). Together with the dietary regulation of EE peptides illustrated by some examples above, these findings suggest that, like in mammals, EE hormone release may be modulated in response to nutrient quality or quantity. In animals with a vascular system, peptides secreted from EE cells can enter the bloodstream and reach tissues at a considerable distance, ranging from other cells in the digestive tract to brain centers regulating appetite (Clemmensen *et al.* 2017). While the above-described studies have provided functional evidence for actions of *Drosophila* EE-derived peptides on receptors in remote organs, implying systemic release into the open circulation of flies, practically nothing is known about how EE peptide hormones are secreted through the visceral muscles and basement membranes into the hemolymph. As well as modulating EE peptide release, nutrient availability can also affect the number of EE cells; signaling through the nuclear hormone receptor Hr96, dietary lipids control EE differentiation during the first few days of adult life, providing another way to couple nutrient availability with tissue architecture and physiology (Obniski *et al.* 2018).

##### Interorgan signaling involving other intestinal cell types

The *Drosophila* gut expresses a battery of receptors for neurotransmitters or peptides not produced by gut-innervating neurons or EE cells (Veenstra *et al.* 2008; Veenstra 2009), suggesting significant modulation of intestinal physiology by systemic signals. Examples of striking systemic effects include the above described control of epithelial turnover by insulin-like peptides or JH, and the coupling of dietary availability of sugars with EC digestive enzyme production via the fat body-derived Activin ligand Daw. Another example is provided by the actions of the diuretic peptide Leucokinin (Lk), secreted into the circulation from CNS-derived nerves that terminate at the abdominal wall (O’Donnell *et al.* 1996; Radford *et al.* 2002; Cognigni *et al.* 2011). Downregulation of either this peptide or its receptor leads to abnormal excreta and extreme fluid retention that can rupture the abdominal wall (Cognigni *et al.* 2011), although a possible contribution of the intestinal Lk receptor remains to be established. Finally, a link between energy balance, intestinal permeability, and immunity has been suggested by the finding that sNPF is a target of the Crtc/CREB energy sensing pathway, and functions to maintain epithelial barrier integrity acting through its receptor in ECs (Shen *et al.* 2016). Although the precise source of sNPF remains to be established, sNPF does not appear to be expressed in the gut (Lee *et al.* 2004), and tissue-specific genetic and expression data points to roles in neurosecretory cells (Shen *et al.* 2016), consistent with roles as a neuroendocrine hormone or in gut-innervating neurons.

Conversely, the gut can also produce long-range signals to affect the physiology of other organs, and can do so using signals other than neurons or EE peptides. A case in point is the production of the signaling protein Hh by larval EC (Rodenfels *et al.* 2014). Circulating Hh regulates developmental timing by controlling ecdysteroid production in the prothoracic gland, and is required for mobilization of fat body TAG stores during starvation (Rodenfels *et al.* 2014). Finally, localized activation of AMPK and/or the autophagy-specific protein kinase Atg1 in the intestinal epithelium or in neurons can remotely affect autophagy, life span, and/or insulin production, but further work will be required to establish the signals involved as well as their physiological significance (Ulgherait *et al.* 2014).

## Conclusions and Outlook

More than a century of using *Drosophila* to study how an animal is made have equipped us with an arsenal of genetic tools that we are now using to explore how *Drosophila* functions. The excitement that the study of the adult fly intestine has sparked is an excellent illustration of how, in only a decade, we have repurposed the powerful genetics of *Drosophila* to make significant discoveries in areas of physiology as diverse as immunity, neurobiology, stem cell biology, and aging. Some of the intestinal mechanisms that we first found in flies have recently been shown to be active in mammals, and may therefore become relevant in the context of human pathologies such as gastrointestinal cancers, aging, or obesity.

In common with many other disciplines (and often biased by historical accidents, laboratory pedigrees, research fashions, *etc*.), our community has focused its efforts on a few topics, such as stem cell biology and aging, and on specific gut portions, cell types, and developmental stages—typically, the intestinal epithelium of two adult midgut regions. Consequently, functions such as digestion and transport, or organs such as the crop and the proventiculus, remain poorly characterized. We may need to delve deeper into the biology of these, not only to shed light on the logic of gut organization and function, but also to investigate what may be their key role in human or insect (patho)physiology.

Looking ahead, what has been our biggest asset may also become our most significant challenge. Indeed, the knowledge that we have so far acquired is effectively a compendium of necessities and sufficiencies: phenotypes arising from genetic screens and gain- and loss-of-function experiments. Although these approaches have proved to be extremely powerful in identifying the effects that certain gene products can have in a particular process, or in revealing how the gut can change and adapt, it is perhaps time to think about how we can best explore physiological drives: what the gut does do and when. What is the relative importance of all the genetic mechanisms that we have found to modulate epithelial turnover? When and how are they deployed? What are the key physiological triggers for specific adaptations or in age-related pathology? Which are the key intestinal sensors? More “holistic” and quantitative approaches may be required to answer these questions, and we may need to integrate spatial and temporal information about genetic events more comprehensively, so that cause and effect can be uncoupled in a physiological context.
